# Emerging Nanotechnology Strategies for Obesity Therapy

**DOI:** 10.1002/advs.202501813

**Published:** 2025-07-14

**Authors:** Weili Chang, Zhaoxu Meng, Yuezhu Zhao, Yake Qi, Zhou Li, He Lian

**Affiliations:** ^1^ School of Pharmacy Shenyang Pharmaceutical University Shenyang 110016 China; ^2^ School of Medical Instrumentation Shenyang Pharmaceutical University Shenyang 110016 China; ^3^ Wuya College of Innovation Shenyang Pharmaceutical University Shenyang 110016 China

**Keywords:** nanomaterials, nanotechnology, obesity, therapy

## Abstract

With the rising prevalence of obesity worldwide, the condition and its associated complications have become a significant public health challenge. The emergence of nanotechnology presents new opportunities for obesity treatment by enabling the design and manipulation of materials at the nanoscale to exhibit unique physicochemical properties, targeted delivery capabilities, and multifunctional therapeutic effects. This review systematically explores recent advances in nanotechnology‐based strategies for obesity management, focusing on the pathological characteristics of obesity, the types of nanomaterials for the treatment, and the therapeutic strategies provided by nanotechnology. Furthermore, the review critically examines translational challenges while proposing innovative solutions. This work aims to foster the effective application and rapid advancement of nanotechnology in addressing obesity.

## Graphical abstract

Three types of nanomaterials used for obesity treatment are highlighted, including inorganic nanomaterials, organic nanomaterials and biomimetic nanomaterials. A detailed and comprehensive introduction to the recent nanotechnology strategies for obesity management is also provided, including drug delivery, photodynamic therapy, hyperthermia, gas therapy, nanoenzyme catalysis, intestinal microbiota regulation and electrical stimulation therapy.

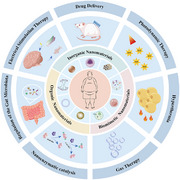

## 1 Introduction

Obesity has emerged as a significant public health issue worldwide. Research indicates that the total number of children, adolescents, and adults living with obesity exceeds 1 billion, and this figure is projected to increase in the coming decades. Obesity is frequently associated with various diseases, including cardiovascular disease, metabolic disorders, respiratory issues, joint problems, and certain types of cancer.^[^ ^1^ ^]^ Obesity affects not only an individual's physical health but also places a significant strain on socioeconomic factors and health‐care systems. Current treatments typically emphasize dietary control, exercise, medications, and surgery. However, the number of approved weight‐loss medications available on the market is limited, and these options often come with undesirable side effects.^[^ ^2^ ^]^ Thus, new and improved treatment strategies are essential to enhance the effectiveness and sustainability of obesity interventions.

Nanotechnology utilizes the unique size‐ and structure‐dependent properties of matter predominantly in the nanoscale (≈1 to 100 nm, as defined in ISO 80004‐1:2023), where quantum effects and increased surface‐to‐volume ratios make interdisciplinary transformative applications possible. The potential of nanotechnology in disease treatment lies in the unique physical and chemical properties of nanomaterials, which have higher surface areas, increased reactivity, and tunable optical properties compared to their microscopic counterparts.^[^ ^3^ ^]^ These characteristics endow nanomaterials with significant potential in disease treatment. For instance, in drug delivery, nanomaterials can carry a larger quantity of drugs and enhance the bioavailability of the medication.^[^ ^4^ ^]^ In recent years, nanomaterials and their delivery systems have garnered considerable attention as an emerging engineering technology in the treatment of obesity. Nanotechnology‐based therapies have shown advantages in various aspects; for example, nanotechnology‐based targeted therapies have made significant progress, and nanomaterials have been used as drug‐delivery vehicles to reduce the systemic side effects of anti‐obesity medications while enhancing treatment safety.^[^ ^5^ ^]^ Additionally, the properties of the materials themselves may provide a range of new advantages that confer specific therapeutic capabilities to nanomaterials, including excellent antioxidant and anti‐inflammatory properties,^[^ ^6^ ^]^metabolic regulatory capacity,^[^ ^7^ ^]^ and the ability to improve the intestinal microbiome, among others.^[^ ^8^ ^]^

**Figure 1:**
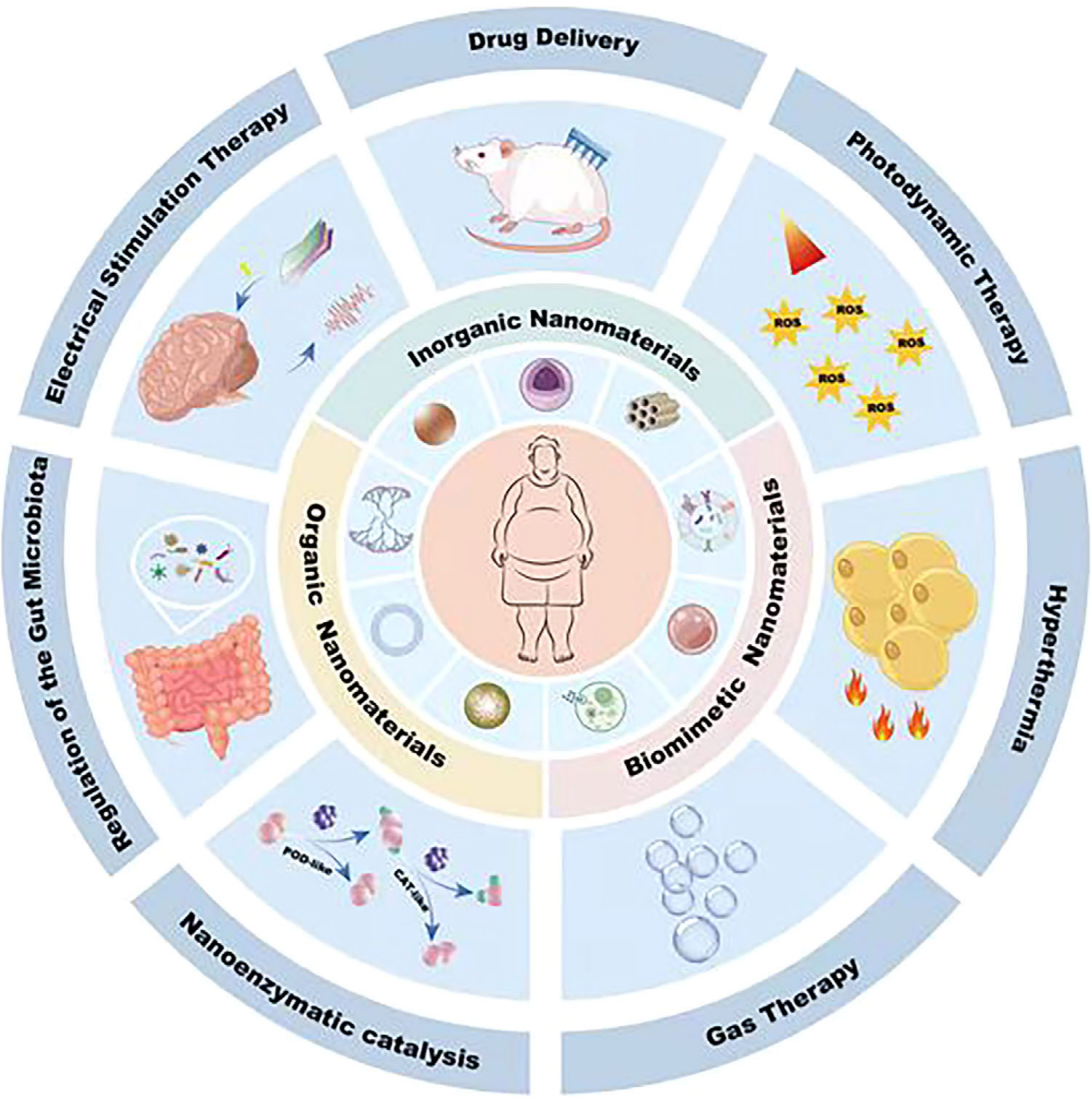
Schematic illustration of the application of nanotechnology in obesity, including the representative classification of reported nanomaterials (including inorganic, organic, and biomimetic nanomaterials), therapy strategies like drug delivery, photodynamic therapy, hyperthermia, gas therapy, nanozyme‐catalyzed therapy, regulation of the gut microbiota, and electrical stimulation therapy.

Herein, we present an overview of the pathological basis and developmental processes of obesity as studied thus far. We also highlight various nanomaterials used in obesity treatment. Furthermore, we offer a detailed and comprehensive overview of recent nanotechnology strategies for the management of obesity (**Figure** **1**). The purpose of this review is to foster a deeper understanding of the research advancements in nanotechnology for the treatment of obesity and to provide valuable guidance for the development of new nanotechnologies aimed at addressing obesity.

## 2 Research Background and Basis for the Application of Nanotechnology in the Treatment of Obesity

**Figure 2:**
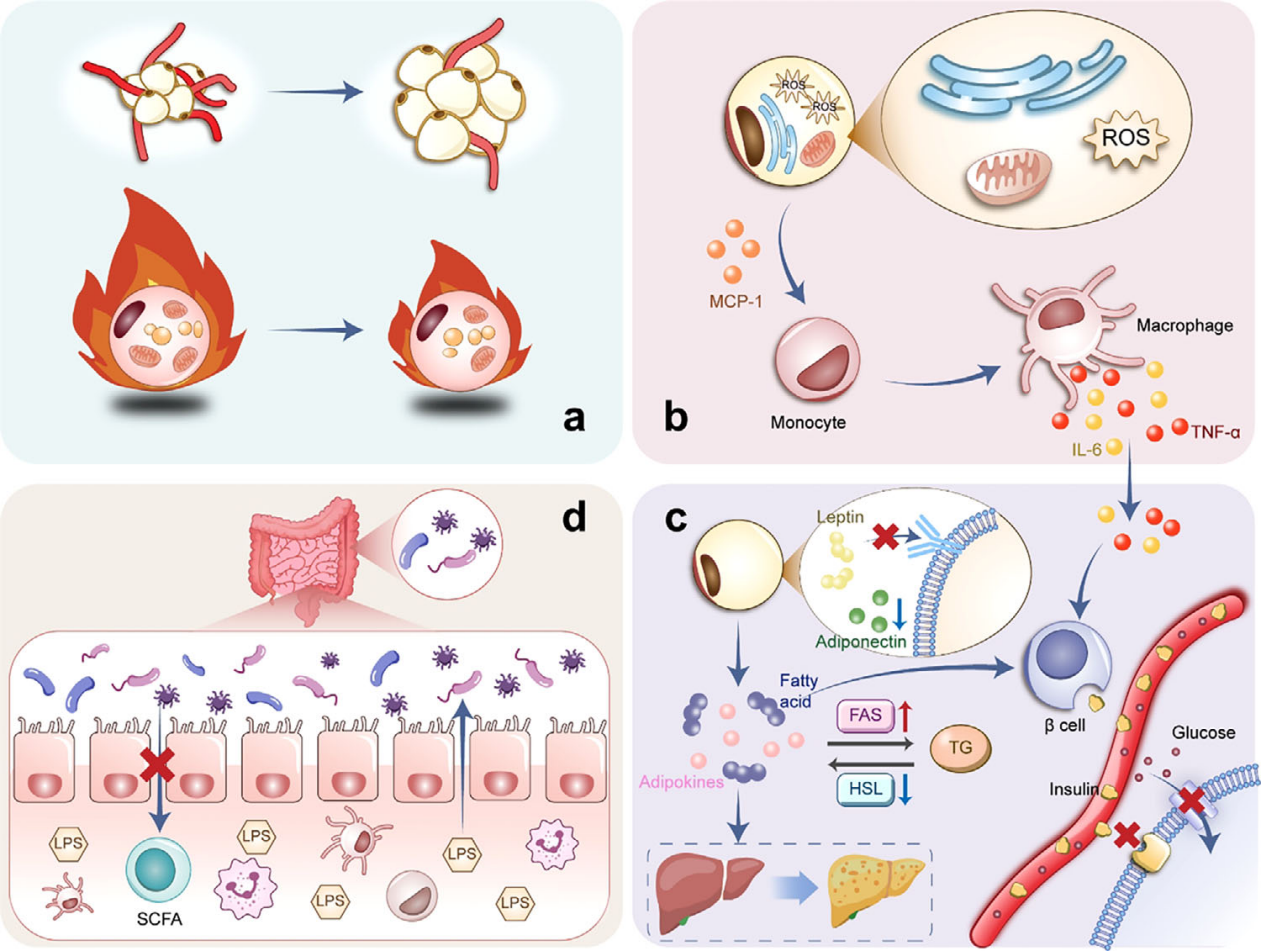
Schematic diagram of the pathological mechanisms related to obesity. a) Hypertrophy and hyperplasia of white adipocytes, accompanied by abnormal angiogenesis (reduced vascular density) in WAT. The suppressed BAT activity leads to diminished thermogenic capacity. b) Mitochondrial dysfunction in adipocytes induces excessive ROS production, triggering oxidative stress. The secretion of monocyte chemoattractant protein‐1 (MCP‐1) recruits monocytes, which differentiate into pro‐inflammatory M1 macrophages. These macrophages release cytokines (TNF‐α, IL‐6), exacerbating adipose tissue inflammation and systemic insulin resistance. c) Leptin resistance and reduced adiponectin secretion disrupt metabolic homeostasis. Elevated free fatty acids (FFAs) and upregulated fatty acid synthase activity promote triglyceride accumulation and induce lipotoxicity. Concurrently, the inhibition of hormone‐sensitive lipase suppresses lipolysis. Excess FFAs accumulate in the liver (steatosis), disrupting insulin signals in peripheral tissues and exacerbating insulin resistance. d) Changes in the composition of gut microbiota reduces the production of short‐chain fatty acid (SCFA) and increases endotoxin levels. This will disrupt the integrity of intestinal barrier, increase permeability, activate immune responses and cause systemic inflammation.

Obesity, as a multifactorial chronic disease, is characterized by intricate pathological processes such as adipose tissue dysfunction, systemic inflammation, and metabolic dysregulation (**Figure** **2**). These mechanisms not only drive fat accumulation but also hinder the efficacy of conventional therapies, which often lack specificity and fail to address the root causes of obesity. The emergence of nanotechnology offers a paradigm shift by utilizing the unique physicochemical properties of nanomaterials (e.g., tunable size, surface modifiability, and biocompatibility) to target these pathological pathways with precision. This section first delineates the key pathological hallmarks of obesity, including adipose hypertrophy, oxidative stress, and gut microbiota imbalance, which collectively form the rationale for nanotechnological interventions. Subsequently, we classify nanomaterials into three categories:inorganic, organic, and biomimetic, and elucidate how their tailored designs (e.g., photothermal conversion, targeted drug delivery, and immune modulation) can directly counteract obesity‐related pathologies, thereby establishing the scientific foundation for the therapeutic applications.

### 2.1 Pathological Features of Obesity

#### 2.1.1 Adipose Tissue Abnormalities

Adipose tissue dysfunction in the obese population is widely acknowledged as a major contributor to the development of obesity‐related comorbidities. Once viewed merely as an inert fat storage organ, adipose tissue is now recognized as an endocrine organ that plays a crucial role in maintaining the internal homeostasis of the body.^[^ ^9^ ^]^ Mammalian adipose tissue comprises three functionally distinct cell types: white adipocytes (WAT), brown adipocytes (BAT), and beige adipocytes. WAT stores energy in unilocular lipid droplets, whereas BAT expends energy through mitochondrial uncoupling protein 1 (UCP1)‐mediated thermogenesis. Beige adipocytes, embedded within WAT depots, can be induced by cold exposure or β‐adrenergic stimulation, exhibiting both lipid storage and thermogenic capacities, making them a critical therapeutic target for obesity.^[^ ^10^ ^]^ Obesity is characterized by WAT hyperplasia, BAT hypoactivity, and impaired beige adipocyte biogenesis, collectively contributing to systemic energy imbalance.^[^ ^11^ ^]^

#### 2.1.2 Oxidative Stress and Inflammation

The interplay between oxidative stress and chronic inflammation constitutes a pivotal mechanism driving adipose dysfunction in obesity. Initially, mitochondrial dysfunction and endoplasmic reticulum stress in hypertrophic adipocytes lead to excessive reactive oxygen species (ROS) production, a hallmark of obese adipose tissue.^[^ ^12^ ^]^ These ROS act as potent signaling molecules, activating pro‐inflammatory pathways such as the NF‐κB and Jun‐N‐terminal kinase JNK through redox‐sensitive transcription factors, which subsequently upregulate chemokines like monocyte chemotactic protein 1 (MCP‐1).^[^ ^13^ ^]^ This chemokine surge recruits monocytes into adipose tissue, where they differentiate into pro‐inflammatory M1 macrophages.^[^ ^14^ ^]^ Activated macrophages secrete cytokines, including tumor necrosis factor (TNF)‐α and interleukin (IL)‐6. These cytokines, on the one hand, inhibit antioxidant defenses (e.g., decreasing superoxide dismutase activity), and on the other hand, enhance the generation of ROS through activation of NADPH oxidases and disruption of the mitochondrial electron transport chain to exacerbate oxidative stress.^[^ ^15^ ^]^ Simultaneously, adipocyte‐derived inflammatory factors such as resistin exacerbate insulin resistance by promoting serine phosphorylation of insulin receptor substrate 1, thereby impairing glucose uptake.^[^ ^16^ ^]^ This reciprocal reinforcement—oxidative stress triggers inflammation, and inflammatory mediators aggravate oxidative damage—creates a self‐sustaining cycle that perpetuates lipid accumulation and metabolic dysfunction.

#### 2.1.3 Endocrine and Metabolic Dysfunction

Critically, inflammatory cytokines and oxidative stress converge to disrupt endocrine homeostasis. Patients with obesity exhibit a multidimensional and complex profile of endocrine and metabolic abnormalities. First, there is an imbalance in the endocrine function of adipose tissue, which secretes a variety of adipokines. In addition to elevated and resistant levels of leptin, there is typically a decrease in the secretion of lipocalin, a substance known for its beneficial metabolic effects, such as improved insulin sensitivity and anti‐inflammatory properties, and the inadequate secretion of lipocalin weakens metabolic regulation.

Insulin resistance refers to a reduced efficiency of insulin in promoting glucose uptake and utilization in the body. In the state of obesity, the lipolysis of adipocytes in adipose tissue releases a significant amount of free fatty acids and various adipokines, prompting macrophages to produce numerous inflammatory factors. This cascade inhibits insulin signaling, ultimately leading to insulin resistance.^[^ ^17^ ^]^ Insulin resistance further reduces the ability of insulin to inhibit lipolysis, resulting in an increased flow of free fatty acids from adipocytes to other tissues. Abnormalities in lipid metabolism are particularly pronounced in patients with obesity.^[^ ^18^ ^]^ The number of adipocytes increases and their size enlarges, thus undergoing hypertrophy; further, the activity of fat synthesis‐related enzymes, such as fatty acid synthase, is enhanced, resulting in an increase in triglyceride synthesis. Concurrently, the activity of key lipolysis enzymes, such as hormone‐sensitive lipase, is inhibited, leading to a decrease in lipolysis. Excess free fatty acids not only accumulate in the liver, contributing to the development of fatty liver, but also interfere with insulin signaling in the liver and peripheral tissues, worsening insulin resistance. Furthermore, they promote inflammation, affect vascular endothelial function, and increase the risk of cardiovascular disease.^[^ ^19^ ^]^

#### 2.1.4 Imbalance of Intestinal Flora

Notably, the metabolic‐inflammatory milieu directly impacts gut ecology. Gut flora plays a significant role in obesity. Research has shown that the gut microbiota of individuals with obesity tends to exhibit lower diversity,^[^ ^20^ ^]^ which means that the microbial species and community structure in the gut are more homogeneous. This imbalance may exacerbate obesity through energy metabolism, inflammation, and gut‐brain axis regulatory mechanisms.

For instance, short‐chain fatty acids (SCFAs, e.g., butyrate) produced by microbial fermentation of dietary fiber directly stimulate intestinal L‐cells to secrete glucagon‐like peptide‐1 (GLP‐1) and peptide YY, thereby suppressing appetite and prolonging satiety.^[^ ^21^ ^]^ A high‐fat diet‐induced depletion of beneficial genera such as *Bifidobacterium* spp. and *Lactobacillus* spp. may impair this regulatory axis by reducing SCFAs production.^[^ ^22^ ^]^ Concurrently, overgrowth of Gram‐negative bacteria (e.g., *Enterobacteriaceae* spp.) releases endotoxins like lipopolysaccharide (LPS), which compromise intestinal barrier integrity, enter systemic circulation, and activate the Toll‐like receptor 4 signaling pathway, triggering chronic low‐grade inflammation and insulin resistance.^[^ ^23^ ^]^ Furthermore, microbial metabolites (e.g., tryptophan derivatives) act on the hypothalamic arcuate nucleus via the vagus nerve or bloodstream, inhibiting orexigenic neuropeptide Y expression while stimulating anorexigenic α‐melanocyte‐stimulating hormone release, ultimately reducing food intake.^[^ ^24^ ^]^ These mechanisms collectively delineate a network through which the gut microbiota systemically modulates energy balance via metabolic, immune, and neural pathways.

The complex pathological characteristics of obesity necessitate the development of more precise and efficient treatments with fewer side effects, as traditional methods often struggle to achieve the desired therapeutic outcomes. Nanomaterials, with their unique advantages, such as small size, large specific surface area, and strong modifiability, can be tailored to address the pathological features of obesity, leading to more effective treatment options. Below, we discuss the application of nanomaterials in the field of obesity treatment.

### 2.2 Nanomaterials for the Treatment of Obesity

**Figure 3:**
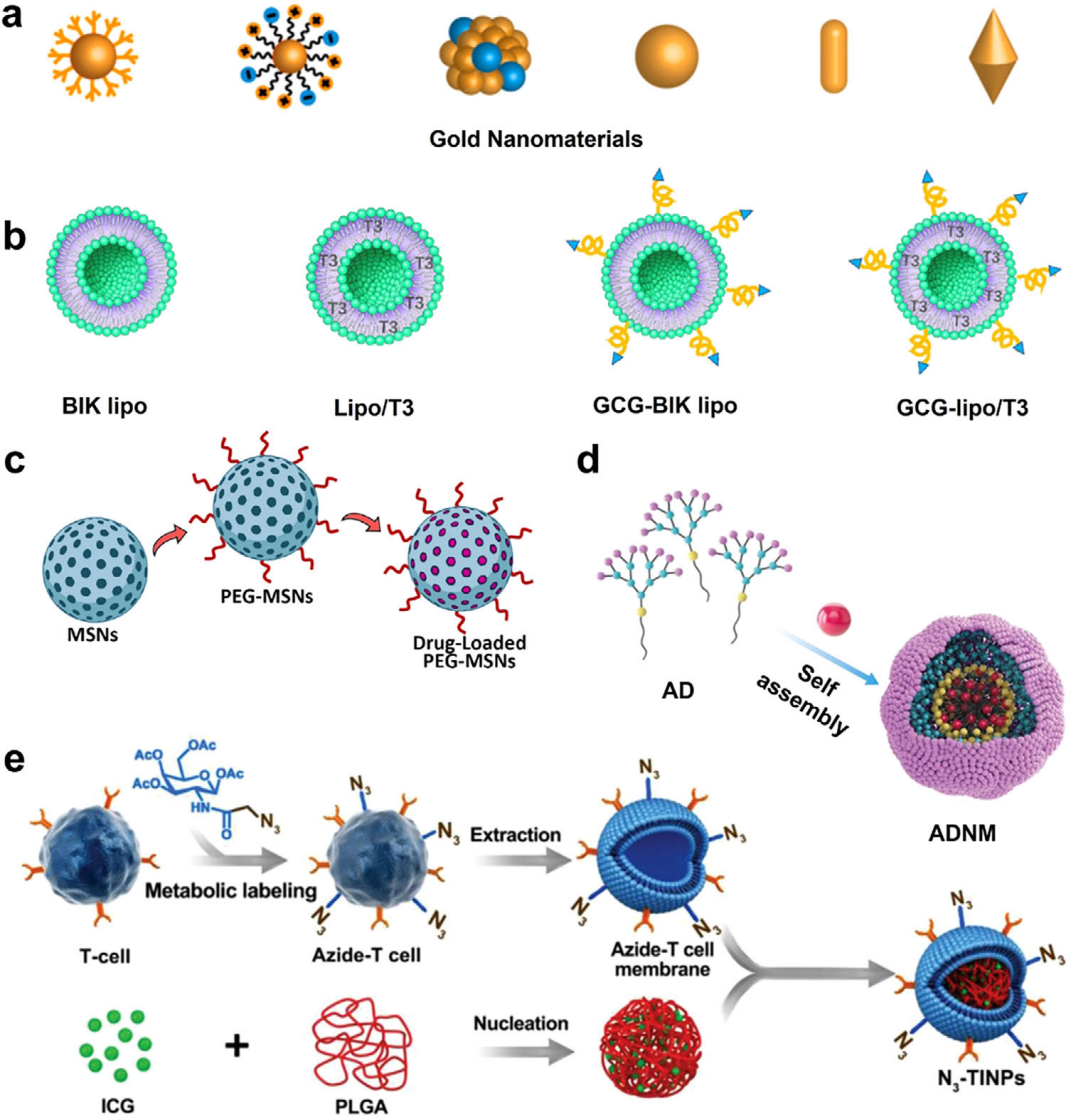
Representative schematic diagrams of typical nanomaterials such as a) AuNPs, b) liposomes, c) MSNs, d) polymeric nanomaterials, and e) biomimetic cell membrane‐coated nanomaterials. a) Reproduced with permission.^[^ ^110^ ^]^ Copyright 2022, American Chemical Society. b) Reproduced with permission.^[^ ^111^ ^]^ Copyright 2023, Elsevier. c) Reproduced under terms of the CC‐BY license.^[^ ^112^ ^]^ Copyright 2022, The Authors, Published by MDPI, Basel, Switzerland. d) Reproduced under terms of the CC BY‐NC‐ND license.^[^ ^113^ ^]^ Copyright 2023, The Authors, Published by PNAS. e) Reproduced with permission.^[^ ^114^ ^]^ Copyright 2019, Wiley.

Currently, the main nanomaterials and their functions used for the treatment of obesity include: 1) Inorganic nanomaterials (e.g., gold nanoparticles, zinc oxide nanoparticles) directly intervene in fat metabolism through photothermal conversion, antioxidation, and other physicochemical properties. 2) Organic nanomaterials (e.g., cationic dendrimer polymers, polyphenol‐based nanomaterials) possess both drug‐targeted delivery and intrinsic biological activities (e.g., anti‐inflammation, intestinal flora regulation). 3) Biomimetic nanomaterials (e.g., cell membrane‐coated nanoparticles, exosomes) utilize natural bio‐interfacial properties to achieve efficient targeting and immune escape. In this section, we will discuss the commonly used nanomaterials in obesity treatment systems and summarize their specific classification, functional applications, and advantages and disadvantages, as depicted in **Table** **1** and **Figure** **3**.

**Table 1:** Nanomaterials for nanotechnology applications in obesity treatment.

Types	Nanomaterials	Characterization	Application	Advantages	Disadvantages	Refs.
Inorganic nanomaterials	AuNPs	Photothermal conversion capability, converting absorbed near‐infrared light into thermal energy	For photothermal lipolysis to induce thermally mediated death of adipocytes	‐ High photothermal efficiency ‐ Biocompatibility ‐ Easy surface functionalization	‐ Potential hepatorenal toxicity ‐ High production cost	[75]
	AgNPs	Anti‐inflammatory activity	Alleviating obesity‐associated systemic low‐grade inflammation	‐ Broad‐spectrum anti‐inflammatory effects ‐ Low immunogenicity	‐ Long‐term accumulation risks ‐ Limited targeting specificity	[87]
	ZnO NPs	Promoting redox homeostasis and reducing mitochondrial oxidative stress disorders	Reducing inflammation to aid weight loss treatment	‐ Strong antioxidant properties ‐ Cost‐effective synthesis	‐ Potential cytotoxicity at high doses ‐ Poor biodegradability	[116]
	Superparamagnetic iron oxide nanoparticles (SPIONs)	SPIONs generate heat in an alternating magnetic field	Generation of heat in magnetic heat therapy for localized fat reduction	‐ Deep tissue penetration ‐ Controllable thermal output	‐ Requires specialized equipment ‐ Risk of iron overload	[117]
	Mesoporous silica (mSiO2)	Large specific surface area, high drug loading, porous structure	Targeted drug delivery, regulation of lipid metabolism	‐ High drug loading capacity ‐ Tunable pore size	‐ Limited in vivo stability ‐ Potential silica‐induced inflammation	[118]
	Polydopamine nanoparticles (PDA)	Near‐infrared light input converted to precisely controlled temperature output	Promotes efficient localized thermal therapy of white fat at mild temperatures, promotes browning of beige fat, and improves obesity and metabolic homeostasis	‐ Excellent photothermal stability ‐ Natural adhesion properties	‐ Complex synthesis process ‐ Limited penetration depth in dense tissue	[119]
Organic nanomaterials	Liposomes	Biocompatible, can encapsulate hydrophilic and hydrophobic drugs	Improve water solubility and stability of the drug and increase bioavailability	‐ High biocompatibility ‐ Versatile for hydrophilic/hydrophobic drugs	‐ Short circulation half‐life ‐ Susceptible to oxidation	[55, 120]
	Recombinant high‐density lipoprotein (rHDL)	Naturally occurring lipoprotein complex with high biocompatibility and natural targeting ability	Ideal drug carrier for encapsulating drug‐targeted delivery to adipose tissue	‐ Innate targeting ability ‐ Low immunogenicity	‐ Limited drug loading capacity ‐ Complex preparation	[67, 121]
	Polylactic acid‐hydroxyacetic acid copolymer (PLGA)	Biocompatibility, biodegradability, surface modifiability	Slow‐release drug delivery prolongs drug action time and improves adipose tissue targeting	‐ FDA‐approved material ‐ Tunable degradation rate	‐ Acidic degradation byproducts ‐ Burst release in the initial phase	[58, 59, 122]
	Polyamidoamine dendrimer (PAMAM, P‐G3)	With a high positive charge density	Selectively distributes in negatively charged visceral fat, inhibiting the storage of unhealthy lipids in “overloaded” fat cells	‐ Efficient binding to anionic extracellular matrix ‐ Inhibits lipid storage	‐ Off‐target organ accumulation (e.g., liver) ‐ Potential cytotoxicity	[123]
Biomimetic nanomaterials	Cell membrane‐encapsulated nanomaterials	Good biocompatibility, extended in vivo circulation time, natural targeting	Acts as a drug carrier to improve the specificity of drug delivery to adipose tissue	‐ Immune evasion ‐ Long circulation time	‐ Limited scalability ‐ Batch‐to‐batch variability	[61, 124]
	Exosomes (Evs)	Rich in bioactive molecules, good biocompatibility, and targeting properties	Act as a drug carriers and regulate fat metabolism, inflammation, appetite, and gut flora to aid in healing	‐ Natural targeting ‐ Low immunogenicity ‐ Cross biological barriers	‐ Low yield in isolation ‐ Heterogeneous content	[51, 54, 125]

#### 2.2.1 Inorganic Nanomaterials

Inorganic nanomaterials, primarily composed of inorganic elements (e.g., metals, metal oxides), exhibit good biocompatibility and functionalization properties, making them highly promising for the treatment of obesity. Gold NPs (AuNPs) are among the most widely used metallic nanomaterials in the field of nanomedicine and have the notable advantage of being easily surface functionalized. By modifying the surface of AuNPs with specific targeting molecules, such as antibodies, peptides, or aptamers, drugs can be efficiently delivered directly to adipose tissue. This targeted approach enhances therapeutic efficacy while reducing side effects.^[^ ^25^ ^]^ Another important and remarkable property of AuNPs is their localized surface plasmon resonance,^[^ ^26^ ^]^ which enables intense light absorption when exposed to specific wavelengths of light and the efficient conversion of light energy into heat. At the appropriate temperature, localized surface plasmon resonance can disrupt the cell membranes of fat cells, leading to the release of intracellular lipid droplets, which are subsequently eliminated through the body's metabolic processes.

Other inorganic nanomaterials include silver NPs (AgNPs), which are also notable in the treatment of obesity, given their ability to inhibit the inflammatory response in the body and reduce the release of pro‐inflammatory cytokines.^[^ ^27^ ^]^ Among metal oxides, nano‐metal oxides such as zinc oxide (ZnO) NPs^[^ ^28^ ^]^ also exhibit good antioxidant effects. Superparamagnetic iron oxide NPs can be modulated by an external magnetic field to accurately target adipose tissue.^[^ ^29^ ^]^ The effect is similar to that of photothermal conversion with AuNPs, which can also generate heat in the presence of an external magnetic field.^[^ ^30^ ^]^

Non‐metallic nanomaterials, such as mesoporous silica and carbon‐based nanomaterials, have a wide range of applications in obesity treatment. Mesoporous silica NPs (MSNs) serve as effective drug carriers due to their large surface area, porous network, and biocompatibility.^[^ ^31^ ^]^ They can effectively encapsulate various drugs and protect them from degradation before reaching the target tissues, thereby enhancing drug effectiveness. Moreover, the vast modification possibilities of MSNs allow for the adjustment of their structure and surface properties, facilitating controlled drug release, enhancing local therapeutic effects, and reducing side effects on other tissues.^[^ ^32^ ^]^

Carbon nanomaterials have also been shown to impact lipid digestion and absorption, making them a potential candidate for obesity treatment.^[^ ^33^ ^]^ These materials can address obesity in two ways. First, with their excellent drug‐carrying properties, carbon nanomaterials can efficiently load drugs that regulate appetite, promote fat metabolism, or inhibit adipogenesis either on their surface or within their internal pores. Their high specific surface area allows them to carry a significant quantity of drugs, thereby enhancing therapeutic efficacy while minimizing side effects on other normal tissues.^[^ ^34^ ^]^ Second, the unique physicochemical properties of carbon nanomaterials, such as the photothermal conversion ability of graphene, enable them to produce localized thermal effects under specific light conditions.^[^ ^35^ ^]^ This targets adipose tissue by destroying the structure of fat cells or affecting their metabolic function, which directly contributes to the acceleration of lipolysis and metabolism.

#### 2.2.2 Organic Nanomaterials

Liposomes possess a structure similar to biological membranes, consisting of a phospholipid bilayer. This configuration provides good biocompatibility and fluidity, allowing liposomes to easily fuse with the cell membranes of fat cells and efficiently deliver encapsulated substances into the cells. This delivery mechanism promotes lipolysis and inhibits fat synthesis, contributing to the treatment of obesity. Additionally, the hydrophilic and hydrophobic regions within liposomes can separately encapsulate drugs with different properties. Hydrophilic drugs are encapsulated in the internal aqueous core, while hydrophobic drugs can be loaded in the lipid bilayer. This structural design significantly enhances the drug‐carrying capacity of liposomes.^[^ ^36^ ^]^

Many polymeric NPs (PNPs), such as polylactic acid (PLA) and poly(lactic‐co‐glycolic) acid copolymer (PLGA) NPs, exhibit good biocompatibility. In addition, the surfaces of these PNPs can be easily modified by chemically bonding or physically adsorbing various functional molecules, such as targeting ligands and drug molecules, to meet different biofunctional needs.^[^ ^37^ ^]^ This modification protects the drug from degradation prior to reaching its target, thereby improving its stability and bioavailability.

Furthermore, PNPs can be engineered to specifically target adipose tissue, enabling the precise delivery of drugs to adipocytes. This targeted approach not only enhances the weight loss effect but also minimizes the distribution of the drug in non‐adipose tissues, thereby reducing side effects.^[^ ^38^ ^]^

Dendritic macromolecules (dendrimers) possess a highly branched 3D structure that features numerous terminal functional groups and internal cavities, providing them with a high drug‐carrying capacity. Their internal structure allows for the encapsulation of drugs while permitting precise control over molecular characteristics such as molecular weight, degree of branching, and types of functional groups. This level of precision enables dendrimers to be tailored for specific applications.^[^ ^39^ ^]^

Cationic nanomaterials, such as polyamidoamine dendritic polymers, can not only act as drug carriers but also show significant potential in the treatment of various inflammatory diseases and cancers by neutralizing negatively charged pathogens.^[^ ^40^ ^]^ For instance, third‐generation polyglutamine (P‐G3) has been demonstrated to inhibit visceral obesity, increase energy expenditure, prevent obesity, and alleviate associated metabolic dysfunction by specifically targeting visceral fat through its inherently high charge density.^[^ ^41^ ^]^

Polyphenols are naturally occurring compounds widely distributed in plants, distinguished by their intrinsic biological properties such as biocompatibility, antioxidation, free radical scavenging, UV absorption, metal ion coordination, and other bioactivities. Owing to their abundant catechol or galloyl groups, these molecules enable dynamic and reversible binding to diverse substrates through multiple molecular interactions, making them versatile building blocks for designing therapeutic nanomaterials.^[^ ^42^ ^]^ This unique combination of dynamic binding ability and modular structure has expedited the development of polyphenol‐functionalized nanoarchitectures, which have been utilized to engineer cell surfaces and active molecule carriers for multifunctional therapy.^[^ ^43^ ^]^ For instance, polyphenols (e.g., tannic acid) functionalized with bioactive molecules (e.g., proteins, nucleic acids, viral carriers) form stable nanocomplexes via metal ion (e.g., Fe^3^⁺)‐mediated coordination, enabling surface modification of living cells, endowing them with new functions, such as regulating the activity of immune cells and enhancing their targeting ability.^[^ ^44^ ^]^ Furthermore, the modular design achieved by adjusting metal ion types, polyphenol concentrations, or incorporating functional polymers enables these nanomaterials to integrate multiple functions. For instance, the single‐cell coating composed of tannic acids and ferric ions can enhance the potency of treating bacteria in the gastrointestinal tracts of patients receiving antibiotics therapy while avoiding the negative impacts.^[^ ^45^ ^]^ As excellent carriers, polyphenol‐functionalized nanostructures can not only deliver therapeutic components,^[^ ^46^ ^]^ but also capture endogenous substances, thereby regulating metabolic balance. For example, pH‐responsive plant polyphenol microspheres integrate tea polyphenols and D‐α‐tocopherol, which can dynamically capture and target fat derivatives in the gastrointestinal tract, significantly reducing fat absorption and promoting excretion.^[^ ^47^ ^]^ These innovative systems have demonstrated multi‐dimensional application potential in obesity treatment.

#### 2.2.3 Biomimetic Nanomaterials

In recent years, biomimetic nanomaterials have garnered significant attention as novel drug‐delivery materials. These nanomaterials are primarily composed of NPs and external biomimetic components. They retain the inherent properties of NPs while acquiring characteristics of biological structures such as immune escape, long circulation, and targeted delivery.^[^ ^48^ ^]^ Among these, cell membrane‐encapsulated NPs have gradually gained importance in the treatment of obesity. Cell membranes provide good biocompatibility and stability, allowing them to circulate in the body for extended periods.^[^ ^49^ ^]^ They can also be resurfaced and modified for targeted delivery to adipose tissue.

In addition to intact cell membranes, cells can secrete extracellular vesicles (EVs). According to their delivery properties and pharmacological applications in other pathologies, EVs appear to be valuable candidates for enhancing the specificity of anti‐obesity drugs.^[^ ^50^ ^]^ EVs can accurately regulate the metabolic processes of adipocytes by delivering various signaling molecules, such as proteins and miRNAs, thereby promoting lipolysis, inhibiting fat synthesis, and improving insulin sensitivity to effectively combat obesity and associated glucose metabolism disorders.^[^ ^51^ ^]^

Regarding inflammation, EVs can carry specific substances that modulate macrophage polarization, reduce the release of inflammatory factors, and mitigate the adverse metabolic effects of chronic inflammation associated with obesity.^[^ ^52^ ^]^ As natural carriers with excellent biocompatibility, low immunogenicity, and inherent targeting properties, EVs can efficiently deliver drugs or active molecules to adipose tissues for the treatment of obesity. They can also cross important biological barriers, such as the blood‐brain barrier, and regulate the appetite‐control region of the brain.^[^ ^53^ ^]^ Moreover, EVs secreted by intestinal flora can even be used to regulate host metabolism,^[^ ^54^ ^]^ opening promising new avenues for obesity treatment.

## 3 Nanotechnology in Obesity: Precision Delivery Systems and Multimodal Therapeutic Mechanisms

The physicochemical properties of nanomaterials dictate their therapeutic applications in obesity management. For instance, inorganic nanomaterials (e.g., gold nanoparticles, AuNPs) leverage photothermal conversion for local hyperthermia; Organic nanomaterials (e.g., liposomes) utilize their amphiphilic structure to encapsulate hydrophilic or hydrophobic drugs; and biomimetic systems (e.g., exosomes) achieve targeted delivery through natural homing capabilities. This section elaborates on how these material‐specific properties enable diverse strategies, including targeted precision delivery systems, energy‐mediated therapies, metabolic and microenvironment modulation, biological regulation strategies, and synergistic combination therapies (**Table** **3**).

### 3.1 Precision Delivery Systems

To further reduce the side effects of drugs and increase their circulation time in the body, researchers have innovatively introduced nanocarriers that deliver drugs specifically to their target sites. As a result, the scope of research has expanded from traditional drugs to encompass a variety of small‐molecule modulators, proteins, cytokines, and gene components. Additionally, the design of drug‐delivery nanocarriers has progressively extended to include liposomes, biomimetic nanomaterials, polymers, micelles, and mesoporous structures, among other materials (**Table** **2**).

**Table 2:** Various nano‐delivery systems for the treatment of obesity.

Types	Drug	Mechanism of action	Delivery system	Characterization	Refs.
Inorganic nanomaterials	Rosiglitazone (RSG)	Promotes browning of WAT	Magnetic Nanoparticles (MNP)	Reduce RSG adverse effects by specifically targeting adipose tissue using magnetic fields	[126]
	Hydroxy‐α‐sanshool		Mesoporous silica nanoparticles modified with adipose‐targeting peptides	Improved stability and targeting of hydroxy‐α‐sanshool	[127]
Organic nanomaterials	Rosiglitazone (RSG)		Cationic Albumin Nanoparticles (cNPs) bound to injectable pluronic F127 thermosensitive hydrogel	Target delivery of RSG to white adipocytes to induce browning while hydrogels create subcutaneous reservoirs to control and prolong the release of cNPs	[128]
			Poly(lactic‐co‐glycolic acid) / polyvinyl alcohol (PLGA/PVA) nanospheres	Specifically deliver RSG to macrophages, where it is released in their acidic environment to reduce the inflammatory response	[129]
			Nanoparticles modified with prohibitin‐targeting ligand and octopamine peptide	Minimize the off‐target effects of RSG and maximize its browning activity through dual‐targeting modifications	[130]
	Resveratrol		Lipid nanocarriers and liposomes	Increases the water solubility and stability of Resveratrol and enhances browning of WAT	[55]
			Temperature‐sensitive hydrogel containing rHDL	Regulate adipocyte metabolism self‐consciously and target it naturally as done by rHDL; disperse rHDL@Resveratrol in temperature‐sensitive hydrogels, and couple this with modulation of its degradation and drug‐release rates to facilitate the sustainable release of the drug into localized adipose tissues over a longer period	[67]
	Mirabegron (MIR)		PLGA microspheres	Successfully mitigate unnecessary cardiovascular risks through local Mirabegron‐loaded PLGA microspheres (MIR‐MS) injections	[58]
	Triiodothyronine (T3)	Increases energy expenditure and promotes fat burning by increasing metabolic rate	Glucagon‐modified liposomes	Target delivery of T3 to adipose tissue reduces its toxicity to non‐adipose tissue	[111]
			Liposomes modified with lipid‐ homing peptide		[57]
	Orlistat	Inhibits gastrointestinal lipase activity and reduces fat absorption	Lipase‐sensitive amphiphilic copolymer BTTPFN‐g‐PCL coupling polymer	Regulate the release rate of orlistat at cyclically varying lipase concentrations	[131]
	Soat2 siRNA		Chitosan‐PLGA nanoparticles	Enhance cellular uptake of siRNA and the positive charge of Chitosan is attracted to the negatively charged intestinal mucosa, allowing it to be retained in the intestine and facilitating siRNA delivery to the intestinal epithelium	[132]
	Metformin	Alleviates insulin resistance, improves sensitivity to insulin, and contributes to weight loss	Soluble PLGA microneedles	Deliver metformin directly to subcutaneous WAT, induce browning, improve energy metabolism, reduce systemic side effects, increase drug bioavailability, and enhance drug penetration through iontophoresis with this delivery system	[64]
	Capsaicinoids	Stimulates sympathetic excitation, enhances brown fat activity, and promotes fat oxidation	Microneedle patches of α‐lactalbumin nanomicelles	Load capsaicin into the hydrophobic core of alpha‐lactalbumin nanomicelles to minimize skin irritation and then deliver it directly to adipose tissue via microneedle patches	[133]
			Adipo‐8 aptamer liposome	Effectively protect nordihydrocapsaicin from adsorption and provide a slow‐release effect with a long residence time in WAT	[56]
	Semaglutide/Liraglutide	Delaying gastric emptying produces a feeling of fullness while acting on the hypothalamus to further suppress appetite	Polymer nanoparticle hydrogels	Significantly prolong the duration of action of semaglutide/liraglutide in vivo	[66]
	Celastrol	Increases leptin sensitivity, decreases appetite, and reduces food intake, resulting in weight loss	Tetrahedral framework nucleic acids (tFNA)	Protect celastrol from enzymatic and other degradation in vivo, prolongs drug circulation time in the body, and improves cellular uptake efficiency	[71]
Biomimetic‐organic composite nanomaterials	Rosiglitazone (RSG)	Promotes browning of white fat cells	Recombinant high‐density lipoprotein (rHDL) based on macrophage membrane (Ma) artifacts modified with P3 peptide	Largely decrease drug leakage and reduce uptake by macrophages with the Ma coating; by decorating Ma with P3 peptide, make the system show superior accumulation in inguinal WAT	[61]
Inorganic‐organic composite nanomaterials	Mirabegron (MIR)		Injectable thermoresponsive hydrogels based on encapsulation with copper sulfide nanodots (CuS ‐NDs)	Ensure the slow release and effective delivery of the drug, and select CuS – NDs as the photothermal agent to produce an efficient synergistic therapeutic effect	[68]
	Metformin	Alleviates insulin resistance, improves sensitivity to insulin, and contributes to weight loss	Hyaluronic acid‐graphene oxide quantum dot nanocomposite	Increase targeting and bioavailability of metformin by targeting CD44 receptors	[134]
	Quercetin	Promote lipolysis, improve fat metabolism, inhibit triglyceride accumulation, and regulate adipokine release	Fluorescent mesoporous silica nanoparticles of polydopamine	Exhibit a well‐aligned mesoporous structure that helps to achieve sustained and controlled release of Quercetin	[69]
	Forskolin		Lipid–silica nanohybrids	Encapsulates Forskolin at a high rate, remains completely in mouse inguinal WAT for at least 5 days, and functions as a reservoir to enable the sustained release of Forskolin in the adipose depot	[70]

#### 3.1.1 Nanocarriers

##### Liposomes

Liposomes are widely used in drug delivery due to their non‐toxic structure, excellent biocompatibility, easy biodegradability, and protective effects on drugs. Resveratrol, a naturally occurring polyphenolic compound found in grape skins, grape seeds, and various foods, is known to regulate adipocyte differentiation, promote fatty acid oxidation, and enhance mitochondrial viability. It may also increase energy expenditure by inducing the browning of WAT. However, its low water solubility, low stability, and poor bioavailability limit its application. Zu et al. successfully synthesized biocompatible, biodegradable resveratrol liposomes and lipid nanocarriers, demonstrating that nano‐embedding enhances the water solubility and stability of resveratrol, which in turn facilitates the browning of WAT.^[^ ^55^ ^]^

In addition to enhancing the bioavailability of a drug, liposomes can be modified for targeted delivery to adipose tissue. Capsaicinoids, such as capsaicin, dihydrocapsaicin, and nordihydrocapsaicin, are promising lipogenic therapeutic agents known for their anti‐obesity and anti‐inflammatory effects. However, traditional, direct delivery methods typically result in minimal accumulation in adipose tissue, making direct application challenging. By using the aptamer adipo‐8, known for its good adipocyte‐targeting activity, loaded into liposomes via lipophilic interactions, researchers have created functionalized liposomal adipo‐8 aptamers that effectively target nordihydrocapsaicin to mature adipocytes, serving as lipid‐lowering factors and thermogenic activators. This approach protects nordihydrocapsaicin from adsorption while demonstrating great potential for encapsulating, transporting, and releasing molecular cargo in WAT .^[^ ^56^ ^]^

**Figure 4:**
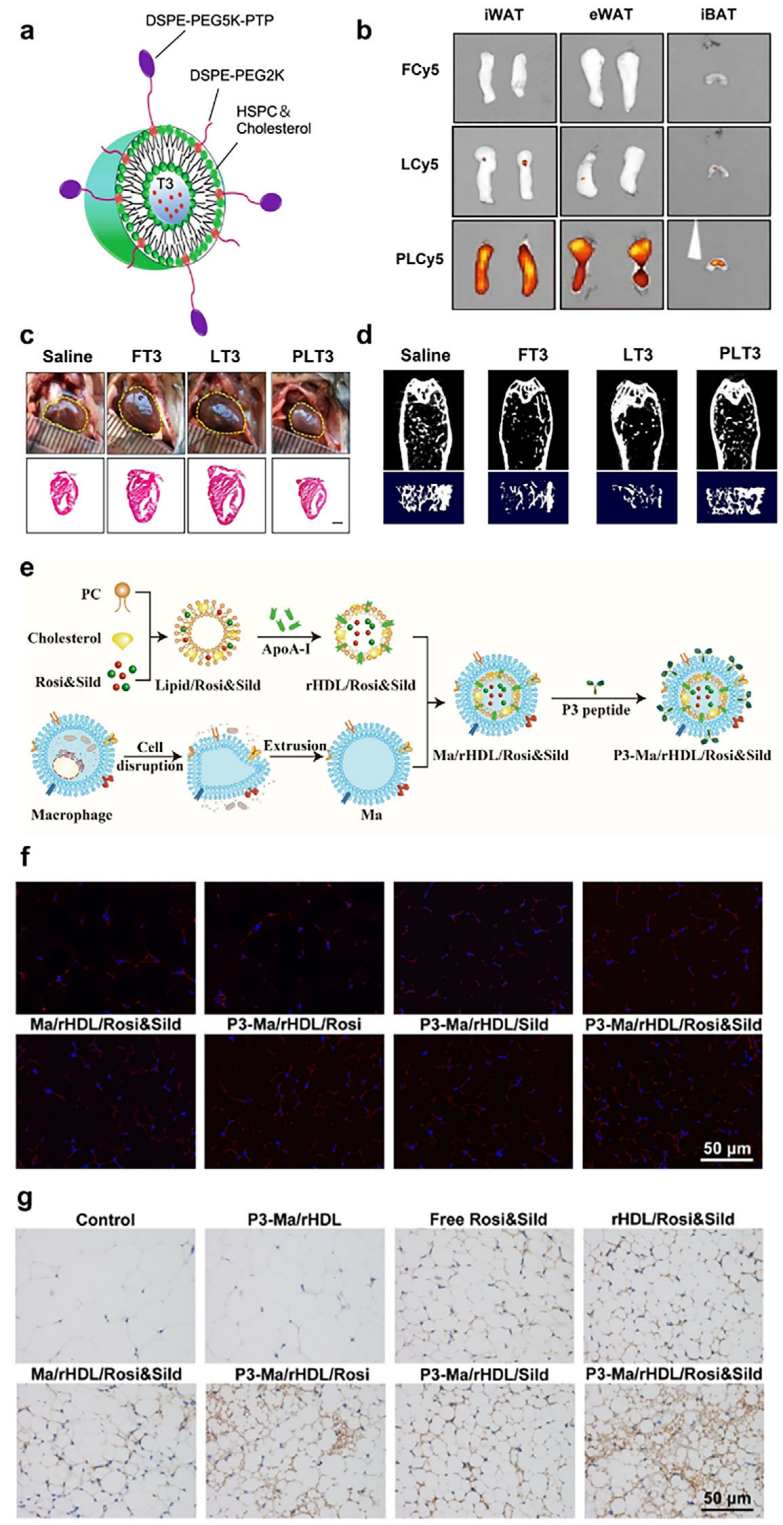
a) Schematic diagram of the structure of triiodothyronine (T3)‐encapsulated PTP‐modified liposomes (PLT3). b) 8‐week‐old male C57BL/6 N mice were injected intraperitoneally (IP) with free Cy5 (FCy5), Cy5‐encapsulated unmodified liposomes (LCy5), or Cy5‐encapsulated liposomes modified with different molar ratios of PTP (PLCy5s) and sacrificed 8 h after injection to observe their ex vivo fluorescence imaging in different adipose tissues. c) Representative images of gross appearance and H&E‐stained cross‐sections of the hearts in male C57BL/6 N mice after 32 days of supplementation with FT3, LT3, PLT3, or saline were measured. d) Representative micro‐CT images of the distal femur (upper panel) and trabecular bone (lower panel) in male C57BL/6 N mice were treated with different forms of T3 or saline for 32 days. Reproduced under terms of the CC‐BY license.^[^ ^115^ ^]^ Copyright 2022, The Authors, Published by Springer Nature. e) Macrophage membrane mimicking nanoparticles (P3‐Ma/rHDL) construction process co‐loaded with Rosi (PPARγ agonist) and Sild (PDE5 inhibitor). f) Representative confocal images of CD31‐positive capillaries in iWAT after treatment with different formulations. g) Representative brightfield images of immunohistochemical staining of UCP1 in iWAT after treatment with different formulations. Reproduced with permission.^[^ ^61^ ^]^ Copyright 2022, Elsevier.

Thyroid hormone (the active form T3) is another natural and potent compound that influences energy expenditure, cholesterol metabolism, and fat oxidation. However, it can cause serious side effects, including tachycardia, heart attack, muscle wasting, and osteoporosis. Moreover, systemic T3 administration has been ineffective in promoting thermogenesis in BAT and WAT due to feedback suppression of sympathetic innervation. In this context, triiodothyronine (T3) is selectively delivered to adipose tissues by encapsulating it in liposomes modified with an adipose‐homing peptide (PLT3) (**Figure** **4**a).^[^ ^57^ ^]^ PLT3‐modified liposomes exhibit excellent properties, including uniform nanoscale size, good physicochemical stability, and appropriate drug‐release behavior. Importantly, PLT3 facilitated the selective enrichment of T3 in adipose tissues (Figure 4b), minimizing the adverse effects of T3 on other tissues. Compared to systemic administration of T3, PLT3 demonstrated superior therapeutic effects in ameliorating obesity and correcting hyperlipidemia and hyperglycemia without causing adverse cardiac effects and skeletal toxicity (Figure 4c,d).

##### Polymer Nanomaterials

Polymeric nanomaterials are widely used in drug delivery due to their tunable size, ease of surface modification, and controlled drug release capabilities. PLGA microspheres containing the selective β3 adrenergic receptor agonist mirabegron (MIR) have been developed.^[^ ^58^ ^]^ MIR‐loaded PLGA microspheres effectively activate BAT and enhance the thermogenic program in WAT. Importantly, localized injection of MIR‐loaded PLGA microspheres successfully mitigated unwanted cardiovascular risks compared to MIR‐treated mice.

Furthermore, PEG‐PLGA NPs were incorporated, resulting in aptamer‐coupled, rhodopsin‐loaded NPs, which were spherical with an average particle size of 146.7 ± 27.85 nm and exhibited a drug loading capacity of ≈6.8% along with slow‐release properties. Confocal microscopy and flow cytometry analyses revealed a significant increase in the internalization of these NPs in differentiated 3T3‐L1 cells compared to rhodopsin‐loaded NPs functionalized with a non‐specific aptamer. This targeted drug delivery technique leverages the targeting advantages of DNA aptamers and the controlled drug‐release properties of PNPs, resulting in a significant increase in therapeutic efficacy while reducing the side effects of the loaded drug.^[^ ^59^ ^]^

##### Bio‐Nanomaterials

Bio‐nanomaterials retain some of the fundamental structure and characteristics of cells, allowing them to encapsulate NPs, increase biocompatibility, reduce immunogenicity, and enhance targeting. Low‐grade inflammation is a negative aspect of obesity, with the stress response triggered by nutrient overconsumption further exacerbated by macrophage infiltration into adipose tissue, leading to systemic metabolic disturbances and imbalances in glucose homeostasis. Focusing on inflammatory targeting could improve the specificity of drug delivery to adipose tissue while alleviating the inflammatory stress associated with anti‐obesity therapy.

Macrophage membranes inherit the surface protein profile and bio‐interface properties of their source cells, providing a protective barrier that shields synthesized NPs from phagocytosis by immune cells. Simultaneously, the natural characteristics of macrophage membranes allow them to accurately recognize antigens and target inflamed tissues.^[^ ^60^ ^]^ An example of a drug delivery platform based on macrophage membrane camouflage involves a recombinant high‐density lipoprotein (rHDL) system modified with a P3 peptide for targeted drug delivery to adipose tissue (Figure 4e). This platform co‐delivers rosiglitazone (Rosi), a peroxisome proliferator‐activated receptor‐γ (PPARγ) agonist, and sildenafil (Sild), a phosphodiesterase type 5 (PDE5) inhibitor, achieving a synergistic therapeutic outcome that promotes adipose browning and angiogenesis. CD31 and UCP1 staining were performed to observe blood vessels and BAT markers in inguinal WAT. After 30 days of treatment, the highest expression levels of CD31 and UCP1 were observed in the P3‐Ma/rHDL/Rosi&Sild group (Figure 4f,g). This finding suggests that the P3‐Ma/rHDL/Rosi&Sild system effectively promoted angiogenesis in inguinal WAT and facilitated the browning of WAT. Overall, this constructed delivery system achieved synergistic therapeutic effects and significantly improved the efficacy of obesity regulation.^[^ ^61^ ^]^

#### 3.1.2 Delivery Technologies

##### Microneedles

Transdermal drug‐delivery systems offer non‐invasive methods for drug administration. Among these, microneedle technology provides innovative solutions for chronic metabolic diseases like diabetes and obesity by utilizing a variety of microneedle types. Nanotechnology enhances drug delivery by integrating nanomaterials, such as liposomes and PNPs, with microneedles. These innovative approaches improve patient outcomes through precise drug administration and real‐time monitoring. Lipid nanocarriers in solubilized microneedles can prolong the release of obesity drugs, enhance drug stability and absorption, and thus improve the management of metabolic disorders.^[^ ^62^ ^]^

Chen et al. developed an innovative fast‐dissolving microneedle patch containing Rosi NPs for the treatment of obesity. This approach demonstrated significant effectiveness in obese mice by effectively penetrating the skin and releasing the drug into the dermis, resulting in reduced body weight and fat, improvement in the inflammatory state of obese adipose tissue, and inducing the browning of white adipocytes.^[^ ^63^ ^]^

Furthermore, combining INT with microneedle technology has shown great potential in enhancing treatment outcomes for various diseases. Metformin was delivered to the subcutaneous WAT of obese C57BL/6J mice using soluble PLGA microneedles in conjunction with INT. This study examined the metformin‐induced WAT browning and its subsequent thermogenic effects. Compared to microneedling alone or INT alone, the combination of microneedling and INT demonstrated superior anti‐obesity activity, evidenced by decreased body weight and fat gain, increased energy expenditure, reduced fat pad size, and improved energy metabolism through WAT browning. This microneedle plus INT approach to induce browning in subcutaneous WAT by delivering metformin and other browning agents may effectively combat obesity in a simple and safe manner.^[^ ^64^ ^]^

**Figure 5:**
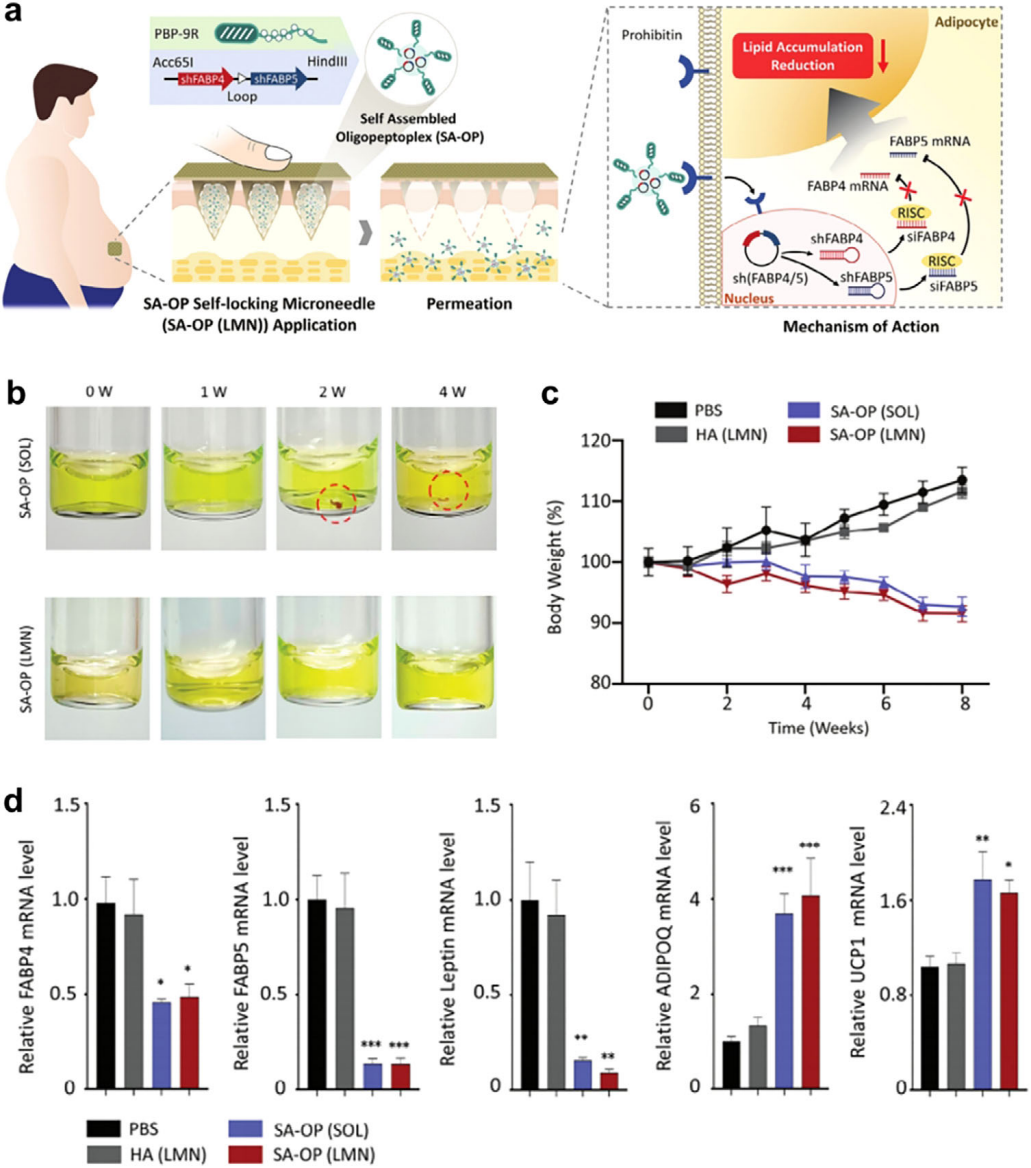
a) Schematic of microneedle patch design encapsulating an oligopeptide‐plasmid complex (SA‐OP). b) Optical observation of SA‐OP aggregation at 4 weeks post‐fabrication. c) Weekly weight changes of rats in each group after administration. d) Relative mRNA levels in vWAT, measured by qPCR and normalized to the mRNA levels of GAPDH. Reproduced with permission.^[^ ^65^ ^]^ Copyright 2024, Wiley.

Additionally, microneedling as a gene delivery system offers an innovative therapeutic tool for the treatment of obesity. Heekyung et al. developed a dissolvable hyaluronic acid‐based self‐locking microneedle (LMN) patch for the precise delivery of self‐assembled genetic material and cationic oligopeptide–oligopeptide complexes (SA‐OP) (**Figure** **5**a).^[^ ^65^ ^]^ These LMNs were designed to encapsulate and deliver plasmid DNA to targeted intracellular organelles by incorporating SA‐OPs. The oligopeptide, featuring both a targeting moiety domain and a positively charged domain, self‐assembled with plasmid DNA encoding dual small hairpin RNAs. Hyaluronic acid serves as a protective matrix for the SA‐OP in the LMN, preventing adverse interactions and aggregation of charged materials due to its negative charge and gel‐forming properties (Figure 5b). In an in vivo evaluation using a diet‐induced mouse model of type 2 diabetes mellitus (T2DM), significant weight loss of 21.92 ± 2.51% and gene silencing in adipose tissue were observed at 6 weeks post‐treatment (Figure 5c,d). These findings suggest that SA‐OP in LMNs serves as a powerful, minimally invasive percutaneous gene delivery platform for the treatment of obesity and its metabolic comorbidities.

##### Hydrogels

The introduction of nanotechnology has significantly enhanced the performance of hydrogel drug delivery systems. For example, nanomaterials have been used to improve the release properties of hydrogels. GLP‐1 receptor agonist drugs are effective for weight loss by reducing hunger, diet, and calorie intake; however, these drugs require frequent administration. To address this, a PNP hydrogel delivery system has been developed. This platform is generated through self‐assembly of dynamic, entropically driven supramolecular interactions between biodegradable nanoparticles (NPs) and hydrophobically modified hydroxypropyl methylcellulose (HPMC) derivatives to develop a long‐acting GLP‐1 receptor formulation.^[^ ^66^ ^]^ Unlike conventional covalently crosslinked hydrogels, PNP hydrogels are formed through strong and dynamic physical interactions, allowing for the sustained, slow release of therapeutically concentrated drug levels, such as liraglutide or simethicone, in rats for up to 42 days after a single injection, which is equivalent to ≈4 months in humans.

Additionally, nanostructures with specific properties can be combined with hydrogels to meet further therapeutic needs. For instance, adipose‐targeted rHDL was encapsulated in a temperature‐sensitive hydrogel drug‐delivery system to achieve targeted delivery of resveratrol to adipose tissues. This approach leverages the natural targeting ability of rHDL to adipose tissues (specifically targeting Scavenger receptor class B type I (SR‐BI) on the surface of adipocytes) along with the hydrogel's capacity to control drug release locally over an extended period. This one‐step obesity treatment strategy effectively targets adipocytes, promotes white adipocyte browning, regulates lipid metabolism, and controls inflammation.^[^ ^67^ ^]^

Similarly, copper sulfide nanodots have been encapsulated in an injectable thermoresponsive hydrogel that exploits their photothermal effect. This hydrogel acts directly on WAT to activate transient receptor potential vanilloid‐1 (the temperature and pain receptor) through heating, inducing white fat browning and promoting lipolysis. Additionally, this therapy combines the delivery of MIR, which significantly improves systemic metabolism, resulting in a powerful synergistic therapeutic effect.^[^ ^68^ ^]^

Beyond the commonly used drug‐delivery platforms mentioned, other nanosystems, such as MSNs and metal NPs, have been explored for anti‐obesity drug delivery in recent years. For example, a fluorescent MSN system coated with polydopamine was developed to efficiently deliver quercetin, inhibiting adipogenesis.^[^ ^69^ ^]^

In addition to using a single nanomaterial for drug delivery, composite nanomaterials possess the general properties of nanomaterials while also exhibiting unique advantages over each individual component, showing potential applications in drug delivery. Lipid‐silica nanohybrids can accommodate the natural supplement trichostatin, converting fat stores into thermogenic adipose tissue by increasing BAT biomarkers. This approach significantly enhances acute glucose uptake, promotes lipolysis, and helps prevent weight gain.^[^ ^70^ ^]^

Furthermore, nucleic acid nanomaterial‐based drug carriers are biocompatible and can be easily programmed and modified. Tetrahedral framework nucleic acids (tFNA) represent a class of nucleic acid nanomaterials with appropriate biocompatibility and tissue and cell permeability. As such, tFNA has been used as a delivery vehicle for ryanodine and celastrol, with suitable stability and in vivo biodistribution. Notably, celastrol‐loaded tFNA simultaneously rescued leptin resistance in the hypothalamus and facilitated energy expenditure in adipose tissue.^[^ ^71^ ^]^

### 3.2 Energy‐Conversion Therapies

Both photodynamic therapy (PDT) and thermotherapy rely on nanomaterials to convert external energy (light/magnetism) into localized therapeutic effects. PDT selectively destroys fat cells by generating ROS through photosensitizers, whereas thermotherapy directly ablates fat or activates metabolic pathways through thermal energy.

#### 3.2.1 Photodynamic Therapy

Photodynamic therapy (PDT) is a non‐invasive strategy for the treatment of obesity that employs laser irradiation at specific wavelengths to excite photosensitizers absorbed by tissues. When in an excited state, these photosensitizers transfer energy to nearby oxygen, generating ROS, which produce cytotoxic effects. PDT represents a promising approach for the non‐invasive treatment of obesity. ROS can induce apoptosis in adipose tissue, effectively inhibiting preadipocyte proliferation and differentiation, and reducing adipocyte numbers. This technique is minimally invasive and highly targeted, thereby reducing damage to surrounding healthy tissues.

**Figure 6:**
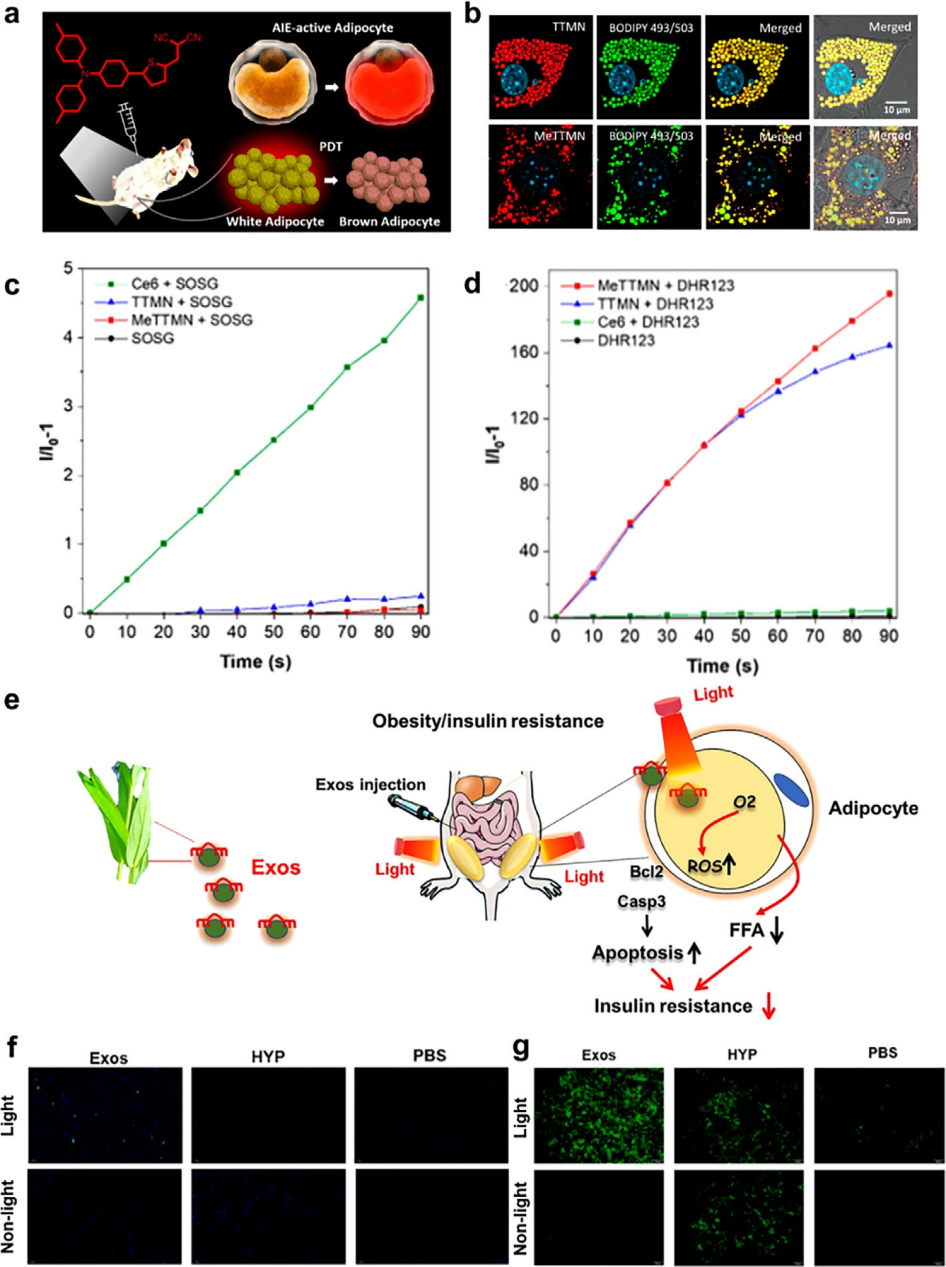
a) Schematic diagram of adipocyte‐targeting type I AIE photosensitizer for photodynamic therapy for obesity via lipid peroxidation. b) TTMN and MeTTMN with the commercial dye BODIPY 493/503 and Hoechst 33258. c) Relative changes in PL intensity of HPF for •OH detection. d) Relative changes in PL intensity of DHR123 for •O_2_^–^ detection. Light power: 10 mW cm^−2^. Reproduced with permission.^[^ ^73^ ^]^ Copyright 2023, American Chemical Society. e) Schematic diagram of *Hypericum perforatum*‐derived exosome‐like nanovesicles (HPExos) for photodynamic therapy in adipose tissue. f) TUNEL fluorescence staining of adipose tissue in the epididymis of HFD mice in each group. g) ROS intensity of mature adipocytes in each group under the same exposure time and background intensity. Reproduced with permission.^[^ ^74^ ^]^ Copyright 2024, Elsevier.

Research on dihydroporphyrin e6 (Ce6)‐based PDT in a high‐fat diet‐induced obese mouse model demonstrated a reduction in lipid accumulation in the liver and a decrease in adipocyte size in ependymal adipose tissue.^[^ ^72^ ^]^ Type I photosensitizers operate effectively in environments with limited oxygen supply, making them particularly suitable for hypoxic PDT treatment, especially in obese adipose tissue characterized by low capillary density and reduced vascular endothelial growth factor, resulting in hypoxia‐like conditions. To exploit this, two red/near‐infrared AIEgens with strong type I PDT capabilities (TTMN and MeTTMN) were used to selectively target adipocyte differentiation and facilitate obesity treatment through photodynamic lipid peroxidation (**Figure** **6**a).^[^ ^73^ ^]^ TTMN and MeTTMN selectively targeted and accumulated in adipocytes, particularly within large lipid droplets (Figure 6b). This targeting ability was attributed to the high hydrophobicity of TTMN and MeTTMN, which promotes hydrophobic–hydrophobic interactions between the AIEgens and the abundant triglycerides and fatty acids found within lipid droplets. The proposed type I AIE photosensitizers generate hydroxyl radicals and superoxide, making them suitable for the photodynamic ablation of excess adipocytes with less reliance on oxygen (Figure 6c,d). Moreover, the ROS produced by TTMN and MeTTMN can initiate a chain reaction of lipid peroxidation, resulting in a synergistic effect that eliminates WAT. In vivo fat reduction studies have demonstrated that the intraperitoneal delivery of AIEgens to adipose tissue is an effective method for PDT. This treatment promotes the browning process of targeted white adipocytes, resulting in fat loss.

Recently, plant‐derived exosome‐like EVs have gained attention due to their similar properties to mammalian exosomes. These vesicles possess a well‐defined lipid bilayer and contain various active molecules, including proteins, nucleic acids, and lipids. Li et al. showed that exosome‐like nanovesicles (HPExos) derived from *Oncorhynchus kimchi* could act as natural photosensitizers, characterized by their intrinsic ability to emit fluorescence and selectively target adipose tissues (Figure 6e). Their findings demonstrated that HPExos‐based PDT resulted in a significant reduction in body weight, triggered a substantial increase in ROS in mature adipocytes, and subsequently induced apoptosis (Figure 6f,g). This treatment significantly downregulated mRNA expression of key genes involved in lipid formation and metabolism, such as *CEBP*, *FABP4*, *PPARG*, and *SREBP*, while markedly inhibiting the proliferation and differentiation of primary adipocytes. These effects contributed to improved glucose metabolism, regulation of adipose cell growth and differentiation, enhanced glucose and lipid metabolic homeostasis, and a reduction in insulin resistance in the context of obesity.^[^ ^74^ ^]^

The utilization of natural photosensitizers derived from plants not only provides ease of access but also offers a cost‐effective solution. Importantly, this approach exhibits low cytotoxicity to healthy tissues and excellent biocompatibility, thereby enhancing its potential as a viable therapeutic intervention.

#### 3.2.2 Hyperthermia

Hyperthermia has a beneficial effect on obesity and metabolic disorders. It directly destroys fat cells and stimulates metabolic processes within them, leading to accelerated lipolysis. Additionally, hyperthermia induces the browning of WAT. However, systemic thermal therapy may increase the risk of neurological and cardiovascular diseases. Nanomaterials present a promising approach for localized thermal therapy given their ability to convert the energy from external physical fields into thermal energy at the lesion site, achieving targeted therapeutic effects. In the context of obesity treatment, thermotherapy primarily includes PTT and magnetic heat therapy.

**Figure 7:**
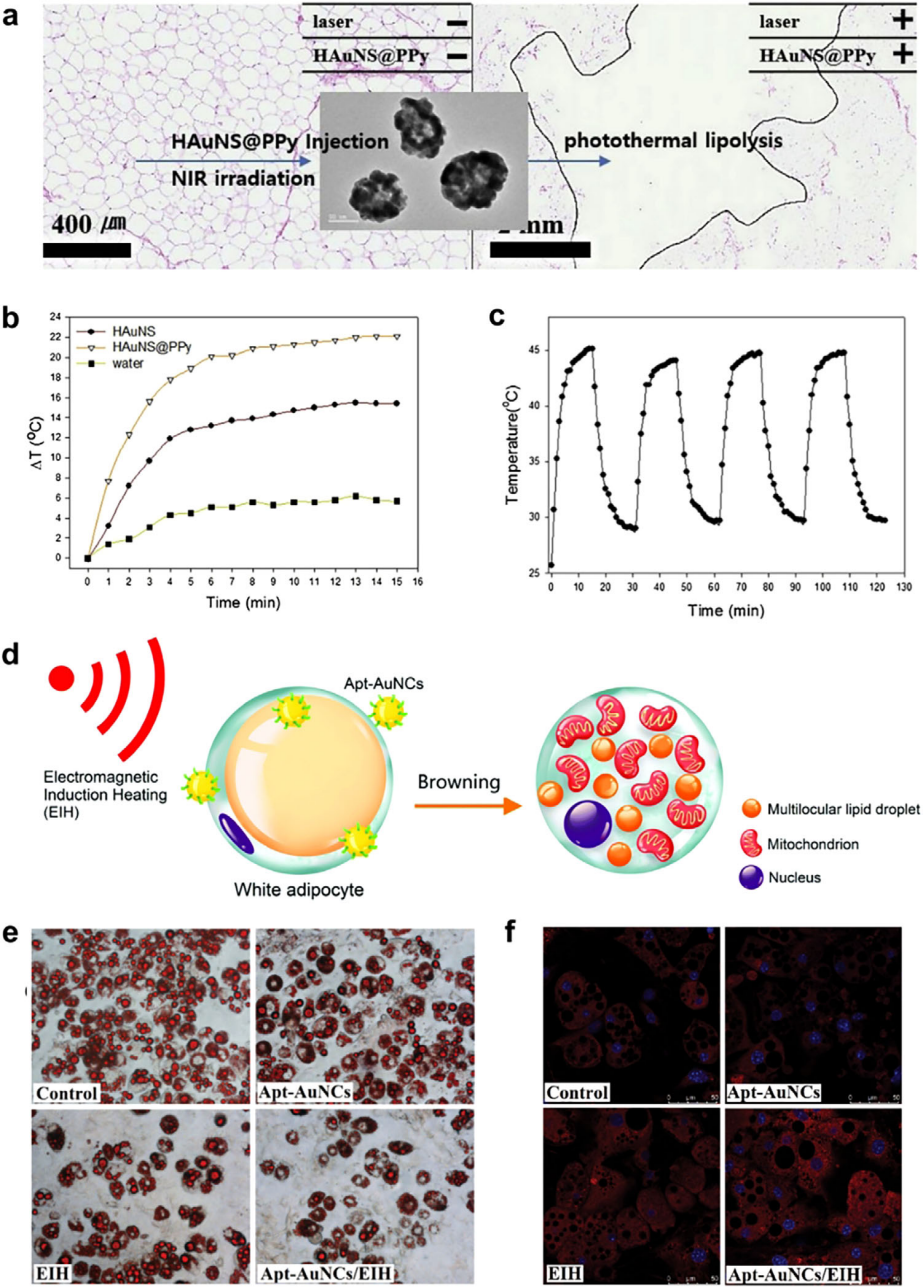
a) Scheme of synthesis procedures for HAuNS@PPy and photothermal lipolysis of porcine subcutaneous fat using NIR laser irradiation. b) Pure water, HAuNS, and HAuNS@PPy solutions at a concentration of 50 µg mL^−1^ under NIR irradiation for 15 min (808 nm, 2 W cm^−2^). c) Temperature changes of HAuNS@PPy solution at a concentration of 50 µg mL^−1^ during four cycles of on/off NIR laser irradiation (808 nm, 2 W/cm^2^). Reproduced with permission.^[^ ^75^ ^]^ Copyright 2024, Elsevier. d) Schematic illustration of browning of white adipocytes by gold nanocluster‐mediated electromagnetic induction heating hyperthermia. e) Distribution and morphology of intracellular lipid droplets in adipocytes by Oil Red O staining. f) Images of mitochondria in adipocytes (the cell nuclei and mitochondria were stained with Hoechst 33342 (blue) and MitoTracker red CMXRos, respectively. Reproduced with permission.^[^ ^77^ ^]^ Copyright 2024, Royal Society of Chemistry.

AuNPs are among the most commonly used agents in PTT. These particles offer several advantages, including low in vivo toxicity, ease of synthesis and functionalization, and the ability to modulate absorbance across the visible to near‐infrared (NIR) spectrum by alterations in their size, shape, and composition. To enhance the photothermal effect, polypyrrole (PPy)‐coated hollow gold nanoshells (HAuNS@PPy) were synthesized as photothermal agents for obesity treatment (**Figure** **7**a).^[^ ^75^ ^]^ The rough surface of HAuNS acts as highly efficient nanoantennae, generating an electromagnetic field that creates multiple intrinsic hotspots within a single NP, thereby enhancing the photothermal effect. PPy, with strong absorbance in the NIR region and a high photothermal effect under NIR laser exposure, further improves the performance of HAuNS, as well as enhancing biocompatibility and photothermal conversion efficiency. As a result, HAuNS@PPy demonstrated a temperature elevation of 6.7 °C higher than that of HAuNS alone (Figure 7b). Histological analyses revealed visible adipocyte necrosis in adipose tissue treated with HAuNS@PPy following NIR laser irradiation. These particles also exhibited excellent photothermal stability through five laser on/off experiments (Figure 7c). Thus, HAuNS@PPy may serve as a potential photothermal agent for the treatment of obesity.

Magnetothermal therapy leverages the unique magnetic properties of magnetite at the nanoscale; these NPs generate heat when subjected to an alternating magnetic field. A composite hydrogel doped with Fe_3_O_4_ microspheres (Fe_3_O_4_‐microspheres@chitosan/β‐glycerophosphate/collagen), referred to as Fe_3_O_4_@Gel, was designed as a magnetic thermotherapeutic agent to promote lipolysis in white adipocytes. Experimental results showed that Fe_3_O_4_@Gel exhibited excellent magnetothermal properties, resulting in decreased lipid accumulation, reduced triglyceride content, and increased mitochondrial activity in WAT cells, all of which contributed to the promotion of lipolysis in these cells.^[^ ^76^ ^]^

The use of thermotherapy to induce browning of WAT is emerging as an attractive treatment for obesity. A new method based on electromagnetic induction heating (EIH) thermotherapy was developed to promote the browning of 3T3‐L1 white adipocytes.^[^ ^77^ ^]^ In this approach, adipocyte‐targeted aptamer‐modified gold nanoclusters (Apt‐AuNCs) served as mediators for EIH (Figure 7d). Apt‐AuNCs exhibit good biocompatibility and excellent targeting properties for white adipocytes. Treatments involving Apt‐AuNCs combined with EIH increased the number of adipocytes displaying characteristic multilocularity and small lipid droplets (Figure 7e), leading to a significant reduction in triglyceride content. Furthermore, Apt‐AuNCs/EIH treatment notably enhanced mitochondrial activity in adipocytes (Figure 7f) and upregulated mRNA levels of key genes involved in browning, including *UCP1*, *PRDM16*, *PPARG*, and *PGC1A*.

### 3.3 Metabolic and Microenvironment Modulation

Active gases (e.g., H₂, NO) and nanoenzymes enable obesity treatment by modulating intracellular redox balance and metabolic microenvironment. Therapeutic gas molecules ameliorate metabolic disorders through anti‐inflammatory and pro‐lipolytic effects, while nanoenzymes mimic natural enzyme activities to scavenge ROS or modulate metabolic pathways.

#### 3.3.1 Gas Therapy

Gas therapy is a novel treatment approach that utilizes therapeutically active gases to regulate physiological functions, reduce inflammatory responses, improve redox status, and influence various biological processes. However, existing gas therapies are limited by their delivery method, including the difficulty in controlling its diffusion within the body, resulting in ineffective concentration at target sites and potential off‐target effects. Furthermore, the lack of precision in gas storage and release mechanisms complicates dose control, making it difficult for traditional delivery systems to achieve stable, spatiotemporally controlled gas release required for sustained therapeutic effects.^[^ ^78^ ^]^

Nanotechnology offers a fresh perspective on gas therapy. Nanomaterials can serve as excellent carriers for gas molecules, allowing efficient loading and protection through innovative design and enabling controllable gas release. For instance, the specific structure and surface properties of NPs can facilitate the precise regulation of gas release rates and locations. Moreover, nanotechnology can enhance the interaction between gas molecules and biological tissues, improving the targeting and bioavailability of gas therapies, thus addressing existing challenges and achieving more effective disease treatment.^[^ ^79^ ^]^

**Figure 8:**
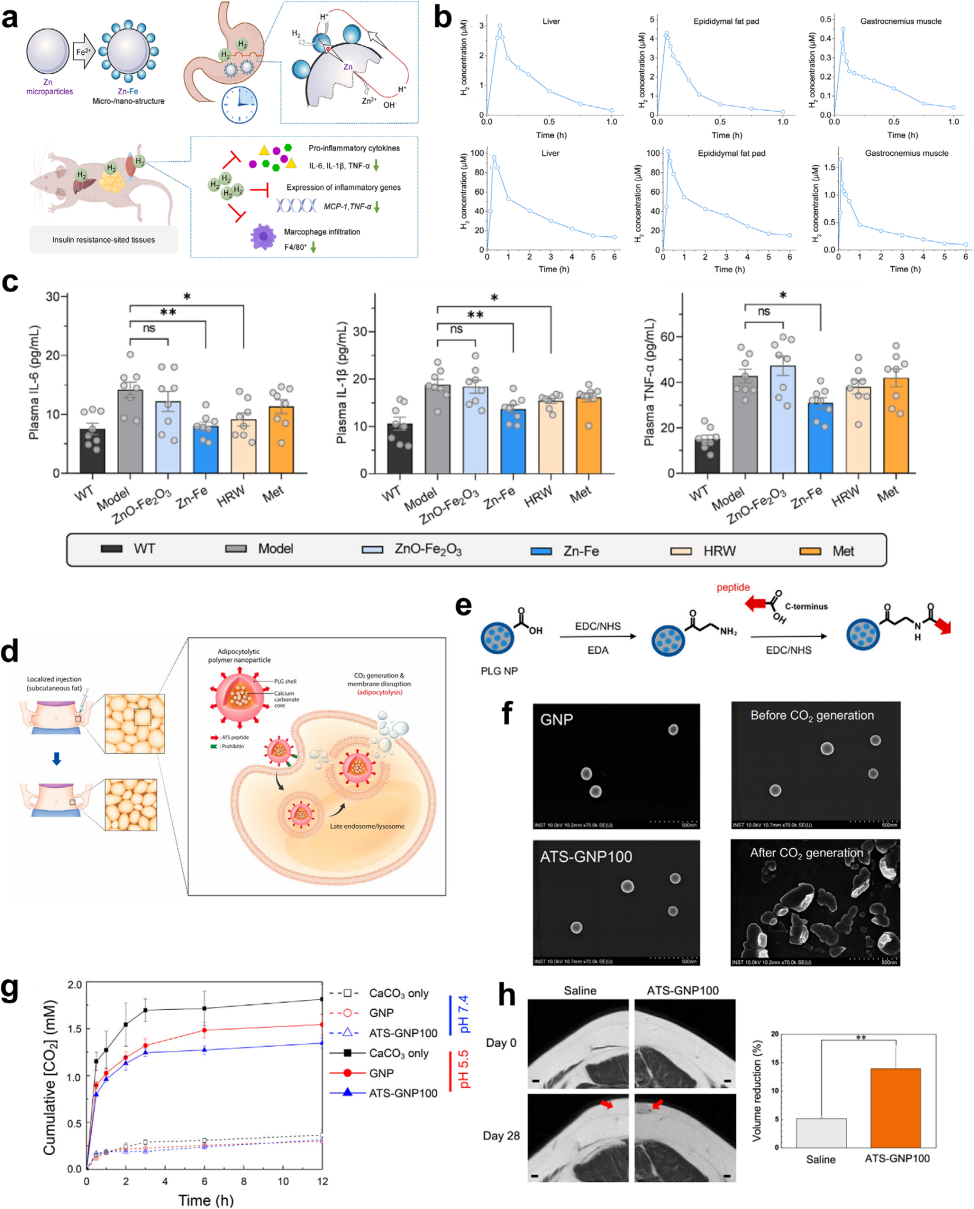
a) Schematic illustration for the structure and construction method of the Zn‐Fe primary‐battery microstructure/nanostructure. b) In vivo monitoring of H_2_ concentration in liver, epididymal fat pad, and gastrocnemius muscle after intragastric administration with micro/nanoparticles with the Fe‐to‐Zn ratio of 1:100 (200 mg kg^−1^) (on) or HRW (800 µM, 10 mL kg^−1^) (behind). c) Sustained H_2_ supply alleviates systemic and local inflammatory response. Plasma levels of IL‐6, IL‐1β, and TNF after various treatments. Reproduced with permission.^[^ ^80^ ^]^ Copyright 2024, Elsevier. d) Illustration for receptor‐mediated endocytosis of an ATS‐GNP to an adipocyte, subsequent intracellular gas generation, and resultant adipocytolysis by membrane disruption. e) Synthetic scheme for covalent conjugation of ATS peptide to the surface of PLG nanoparticles using ethylenediamine as a linker via carbodiimide chemistry. f) Scanning electron microscopic images of GNP, ATS‐GNP100, and ATS‐GNP100 before and after gas generation. g) Release profiles of CO_2_ gas from CaCO_3_ only, GNP, and ATS‐GNP100 at different pH values ([nanoparticle] = 0.5 mg mL^−1^, *n* = 4). h) Magnetic resonance images of the subcutaneous fat layer of the minipig at days 0 and 28 postinjection of either saline or ATS‐GNP100 (scale bar, 1 cm). The arrows indicate injection sites. Fat volume reduction at the injection site was quantified from the image (*n* = 4; Student's *t*‐test; ^**^ *p*<0.01). Reproduced with permission.^[^ ^82^ ^]^ Copyright 2023, American Chemical Society.

Common gases used in therapeutic applications include H_2_ and NO. In the context of obesity, chronic low‐grade inflammation can impair insulin signaling, leading to insulin resistance. H_2_ molecules are safe and effective anti‐inflammatory agents; however, conventional delivery methods struggle to provide high doses and prolonged H_2_ therapy in relevant tissues, resulting in limited therapeutic benefits. To overcome this, a novel strategy has been developed to enhance the hydrolysis rate of Zn by constructing Zn‐Fe primary cell micro/nanostructures and modulating the Zn‐to‐Fe ratio to match the gastric emptying time window. This approach maximizes the bioavailability and therapeutic efficacy of H_2_ (**Figure** **8**a). In vivo monitoring indicated that H_2_ is generated in the stomach and can efficiently accumulate in major insulin resistance‐localized tissues, such as the liver, adipose tissue, and skeletal muscle, at high doses over an extended duration (Figure 8b). Daily oral administration of Zn‐Fe significantly ameliorated obesity‐associated inflammation and effectively improved insulin resistance in an obese diabetic leptin‐deficient (ob/ob) mouse model (Figure 8c).^[^ ^80^ ^]^

NO is a gaseous signaling molecule that plays a critical role in various biological processes, including inflammatory responses, metabolism, cardiovascular function, and cognitive function. NO can stimulate metabolic rate by enhancing mitochondrial biogenesis and activating brown fat. Consequently, a new technique for the delivery of exogenous NO involves the development of a peptide amphiphile nanomatrix gel that releases sustained levels of NO over 1 month (PANO Gel).^[^ ^81^ ^]^ The peptide amphiphile consists of multilayers of hydrophilic NO‐releasing peptide sequences linked to hydrophobic alkyl tails. Its amphiphilic nature enables self‐assembly into cylindrical micellar nanofibers, which are crosslinked by calcium ions to form nanomatrix gels. The unique structure and NO‐releasing peptides in these gels facilitate NO production. Subcutaneous injection of the NO‐releasing nanomatrix gel (PANO gel) led to a reduction in high‐fat diet‐induced weight gain, fatty liver, and pro‐inflammatory responses while enhancing glucose tolerance and insulin sensitivity, likely due to increased lipolysis and browning of WAT.

Lipolysis is a non‐surgical approach for the reduction of localized adipose tissue, while adipocytolysis refers to a cytolytic mechanism that disrupts the plasma membrane, leading to the destruction of adipocytes. CO_2_ may promote membrane disruption in adipocytes. In this regard, a PNP system was designed to prepare CaCO_3_‐loaded PLGA NPs as gas‐producing NPs (GNPs). The adipocyte‐targeting sequence, ATS peptide, was chemically coupled to the surface of the PLGA NPs (ATS‐GNP), facilitating target‐specific endocytosis by adipocytes (Figure 8d,e).^[^ ^82^ ^]^ Endocytosis of these NPs by adipocytes allows for CO_2_ production under acidic pH conditions. Various properties of the NPs were characterized, including their size, morphology, and gas‐release profile (Figure 8f,g). In vitro studies assessed the cytotoxicity of the NPs, and their efficacy as an adipocytolytic agent was evaluated in high‐fat diet‐induced mouse and porcine obesity models. Results indicated that topical injection of these adipocytolytic NPs significantly reduced subcutaneous fat (Figure 8h).

#### 3.3.2 Nanoenzymatic Catalysis

Nanoenzymes possess excellent inherent enzyme‐mimetic properties, making them a promising alternative to natural enzymes. They offer exceptional advantages such as modulation of oxidative stress, ease of storage, adjustable catalytic activity, superior stability, and scalability. Previous studies have shown that excess ROS can lead to oxidative stress, disrupting the function of white adipocytes and directly hindering their respiration, further contributing to obesity. Recently, several nanoenzymes exhibiting significant ROS scavenging capacity have emerged as potent antioxidants with high efficacy in obesity treatment.

Aptamer‐modified atomically precise gold nanoclusters (Apt‐Au_25_NCs) have been used as targeted nanoenzymes to scavenge ROS in white adipocytes.^[^ ^83^ ^]^ These Apt‐Au_25_NCs demonstrated high targeting ability and low cytotoxicity against white adipocytes as well as high superoxide dismutase (SOD)‐like and catalase (CAT)‐like activities in a concentration‐dependent manner, with good thermal and pH stability compared to natural SOD and CAT, effectively scavenging ROS in white adipocytes.

Cerium oxide (CeO_2_) NPs are widely recognized for their ability to participate in diverse cross‐reactions involving ROS and inflammation, modulating the cellular microenvironment by mimicking the activity of several endogenous antioxidant enzymes such as SOD, CAT, and peroxidase. To enhance the activity of CeO_2_, novel nanocomposite nanoenzymes based on mesoporous silica‐coated CeO_2_ (CeO_2_@mSiO_2_) have been developed.^[^ ^84^ ^]^ These nanocomposites have been shown to reduce circulating levels of triglycerides, LDL cholesterol, and palmitic acid while reversing the pro‐inflammatory phenotype of macrophages. They induce macrophage polarization from the M1 phenotype (which relies on glycolysis for energy) to the M2 phenotype (which uses oxidative metabolism). Lipomics and gene expression analyses indicated amelioration of hyperlipidemia as well as hepatic and lipid metabolism dysregulation, associated with the downregulation of the hepatic PI3K–mTOR–AKT pathway and reduced levels of the M1 pro‐inflammatory cytokine TNF. Furthermore, the mSiO_2_ coating maximized the cellular antioxidant effects of CeO_2_ while minimizing non‐hepatic biodistribution, thereby enhancing its in vivo safety.

**Figure 9:**
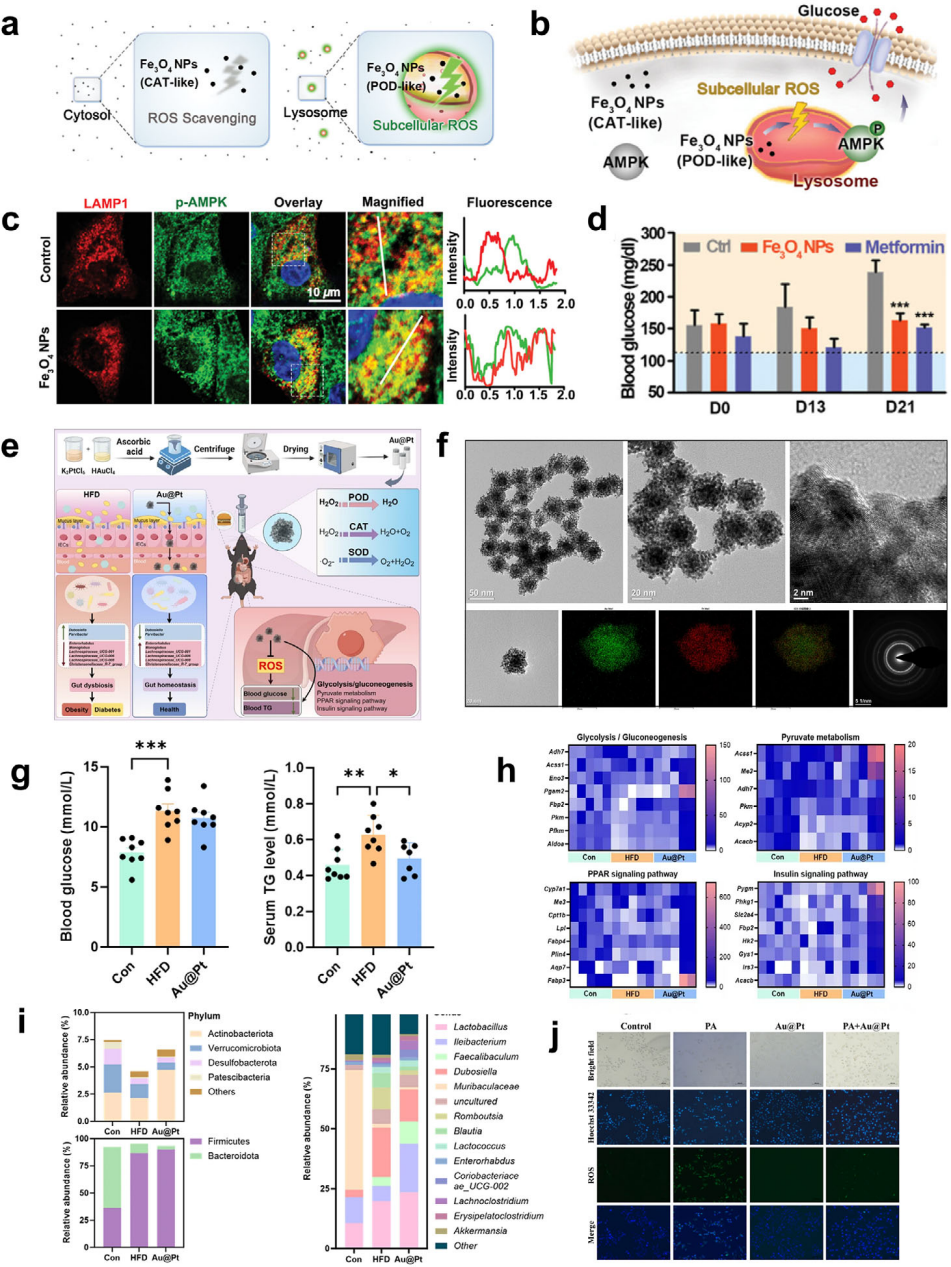
a) Schematic depiction of the organelle‐specific nanozyme activity of Fe_3_O_4_ NPs. CAT: catalase. POD: peroxidase. b) Schematic depiction of the metabolic function of Fe_3_O_4_ NPs in metabolically active cells. c) Colocalization analysis of phosphorylated AMPK (p‐AMPK, green) and lysosomes (LAMP1, red) in L6 myoblast cells after exposure to Fe_3_O_4_ NPs (25 µg mL^−1^). d) Fasting blood glucose levels of ob/ob mice in each group. Reproduced under terms of the CC‐BY license.^[^ ^85^ ^]^ Copyright 2020, The Authors, Published by Wiley. e) Schematic depiction of Au@Pt nanozyme for the modulation of glucose and lipid metabolism. f) TEM images of Au@Pt nanozyme. g) The levels of fasting blood glucose and triglyceride. h) The heat‐map of differentially expressed genes in glycolysis/gluconeogenesis, pyruvate metabolism, PPAR signaling pathway, and insulin signaling pathway. i) The gut microbiota relative abundance at the phylum and genus level. j) The representative fluorescence pictures of ROS (green) and nucleus (blue). Reproduced under terms of the CC‐BY license.^[^ ^86^ ^]^ Copyright 2024, The Authors, Published by Springer Nature.

Additionally, nanoenzymes can regulate metabolic processes by generating appropriate levels of ROS. Fe_3_O_4_ NPs exhibit pH‐dependent dual enzyme activities: under neutral pH conditions, they display CAT‐like activity to scavenge ROS, while under acidic conditions, they exhibit peroxidase‐like activity capable of generating ROS (**Figure** **9**a). Research has demonstrated that Fe_3_O_4_ NPs can locally regulate the energy sensor adenosine 5′‐monophosphate‐activated protein kinase (AMPK) to promote glucose metabolism and insulin response through peroxidase‐like activity in acidic organelles like lysosomes (Figure 9b).^[^ ^85^ ^]^ To investigate this, lysosomes were labeled with a transfected red fluorescent protein‐tagged lysosomal‐associated membrane protein 1 (LAMP1) to map the subcellular localization of phosphorylated AMPK. In the absence of Fe_3_O_4_ NPs, phosphorylated AMPK signals were evenly distributed in the cytosol, with ≈20% localized to lysosomes. Incubation with Fe_3_O_4_ NPs significantly increased activated AMPK levels colocalized with lysosomes (Figure 9c), confirming that the lysosomal ROS generated by Fe_3_O_4_ NPs were sufficient to induce AMPK activation. Administration of Fe_3_O_4_ NPs or metformin every other day over 3 weeks significantly lowered hematological parameters to similar levels (Figure 9d).

Another study developed Au@Pt nanoenzymes, which exhibit multiple enzyme‐like activities and can regulate glucose and lipid metabolism (Figure 9e). The Au@Pt nanozyme, a bimetallic core‐shell structure composed of gold and platinum (Figure 9f), has garnered considerable attention due to its excellent catalytic activity and stability. Specifically, ingestion of Au@Pt nanoenzymes not only enhanced glucose tolerance but also decreased serum triglyceride levels (Figure 9g). Notable changes in the expression of genes related to glucose and lipid metabolism signaling pathways, including insulin signaling, glycolysis/glucuronidation, PPAR signaling, and pyruvate metabolism, were observed following the administration of Au@Pt (Figure 9h). Additionally, 16S rRNA profiling of fecal samples from Au@Pt nanoenzyme‐treated mice revealed significant alterations in the abundance of beneficial intestinal microbiota, closely associated with modified metabolic phenotypes (Figure 9i). Furthermore, Au@Pt effectively eliminated ROS and enhanced CAT and SOD activity in hepatocytes within an inflammatory microenvironment in vitro (Figure 9j).^[^ ^86^ ^]^

### 3.4 Biological Regulation Strategies

Gut microbiota modulation and neuromodulation via electrical stimulation achieve systemic obesity management through biological signaling networks. Gut microbiota regulates the gut‐brain axis via metabolites such as short‐chain fatty acids (SCFAs), suppressing appetite and improving energy metabolism, while neurostimulation directly targets the vagus nerve to modulate hypothalamic feeding centers via electrical signals, thereby reducing caloric intake. These strategies target distinct biological signaling networks, offering multidimensional interventions for obesity treatment.

#### 3.4.1 Regulation of the Gut Microbiota

There is a strong and complex bidirectional association between obesity and gut flora. Obesity can trigger dysfunctions in the composition and function of intestinal flora, leading to an imbalance between thick‐walled bacilli and anabolic bacilli that results in increased energy intake, accelerated fat accumulation, and intensified inflammation. Conversely, disorders of intestinal flora significantly contribute to the development of obesity, with metabolites such as SCFAs and other abnormalities affecting hormone secretion related to appetite and energy metabolism via the gut–brain axis, further exacerbating obesity.

Nanotechnology offers new opportunities for the modulation of gut microbes to treat obesity. AgNPs have demonstrated broad‐spectrum antimicrobial activity and have long been used in biomedical applications as antibacterial and anti‐inflammatory agents. It has been found that polyvinylpyrrolidone (PVP)‐coated nanosilver may serve as a potential modulator of gut microbiota. Administration of nanosilver increased the abundance of *Micrococcus wartsii* and *Acidithiobacillus ferrooxidans* to 3.3% and 0.5%, respectively, while reducing *Epsilonbacteraeota* and *Actinobacteria* phyla to 1.4% (*p*>0.05) and 0.6%. These outcomes confirmed the protective effect of nanosilver against high‐fat diet‐induced gut microbiota dysbiosis in obese mice.^[^ ^87^ ^]^ In addition to AgNPs, other metal nanoparticles, such as chromium (Cr), have shown unique potential in regulating intestinal flora and metabolic disorders. Chromium NPs (CrNPs) may also be used as dietary compounds to combat obesity‐related diseases. The negative effects of a high‐fat diet in the rat colon were mitigated by switching to a low‐fat diet supplemented with CrNPs. Microbiota sequencing analysis revealed that the primary effect of CrNPs was to alter intestinal flora activity. The study also noted that switching from a high‐fat diet to a low‐fat diet significantly increased the production of SCFAs, decreased secondary bile acid levels, and increased microbial diversity and beneficial enzyme activities. Therefore, combining specific modulation by nanomaterials with dietary modification may be an effective strategy for synergistic treatment of obesity.^[^ ^88^ ^]^

Exosome‐like nanovesicles isolated from edible plants have shown diverse activities and a regulatory effect on gut microorganisms. For instance, kidney bean‐derived exosome‐like nanovesicles (KBELNs) were investigated for their effects on high‐fat diet‐induced obesity. Oral administration of KBELNs significantly reduced body weight and liver weight, increased SCFAs, and improved blood glucose and lipid levels in obese rats. This was attributed to KBELNs enhancing the diversity of intestinal flora and ameliorating obesity by regulating the composition of gut microbiota, promoting beneficial bacteria, and improving SCFA levels.^[^ ^89^ ^]^

Additionally, orange peel‐derived exosome‐like nanovesicles (TNV) have been shown to regulate lipid metabolism and gut flora.^[^ ^90^ ^]^ TNVs significantly inhibited insulin resistance in db/db mice, reduced hepatic lipid accumulation, promoted intestinal mucosal repair, rescued dysbiosis of intestinal flora, and regulated colonic SCFA and hepatic bile acid metabolism. Moreover, TNVs restored expression of key genes for glucose and lipid metabolism (*ACC*, *AMPK*, *CD36*, *LXRA*, *PPARG*, *SREBP1*) and activated genes related to glycolysis (*G6PC*, *GLUT2*, *PCK1*, *PEPCK*) in db/db mice. In mechanistic studies, TNVs reduced lipid accumulation in 3T3‐L1 and AML‐12 cells by regulating glucose and lipid metabolism‐related genes (*UCP1*, *FGFR4*, *PRDM16*, *PGC1A*, *Tmem26*, *Cpt1*, *Cpt2*, and *PPARA*).

Furthermore, it has been shown that gut microbiota can be trained to release beneficial outer membrane vesicles (OMVs) via diet‐derived exosome‐like NPs.^[^ ^91^ ^]^ OMVs are nanostructures expelled by bacteria through membrane budding, formed by the bacterial outer membrane containing phospholipids and periplasmic proteins. These EVs are involved in several physiological processes, including enhancing bacterial survival through the detoxification of harmful compounds, biofilm formation, and regulating host–pathogen interactions.^[^ ^92^ ^]^

Akkermansia muciniphila, a mucinophilic probiotic, has been recognized as a promising “next‐generation beneficial microorganism” for the treatment of metabolic disorders such as obesity and T2DM. Kumaran et al.^[^ ^93^ ^]^ found that OMVs released from A. muciniphila in the human gut, when trained with garlic‐derived exosome‐like NPs (GaELNs), reversed high‐fat diet‐induced obesity and T2DM in mice. This was attributed to GaELNs promoting the growth and production of OMVs by A. muciniphila, along with altering the lipid composition of OMVs enriched in phosphatidylcholine, thus enhancing interactions with GLP‐1R and increasing insulin signaling. Moreover, GaELN‐regulated OMVs may suppress high‐fat diet‐induced inflammation by modulating the cGAS–STING pathway in brain microglia, highlighting the role of plant NP‐trained OMVs from gut bacteria in regulating gene expression in the brain and addressing brain dysfunction associated with metabolic syndrome. These findings suggest innovative nanotechnology strategies to improve gut health and metabolic disorders through the modulation of gut microbiota.

#### 3.4.2 Electrical Stimulation Therapy

In exploring multiple obesity treatments, the combination of nanotechnology and electrical stimulation, particularly targeting the vagus nerve, presents a promising new perspective. Pulsed electrical stimulation of the vagus nerve can induce various physiological functions related to food intake, energy metabolism, and glycemic control, resulting in significant weight loss. Thus, in vivo vagus nerve stimulation shows great potential in the regulation of food intake as a treatment for obesity.

**Figure 10:**
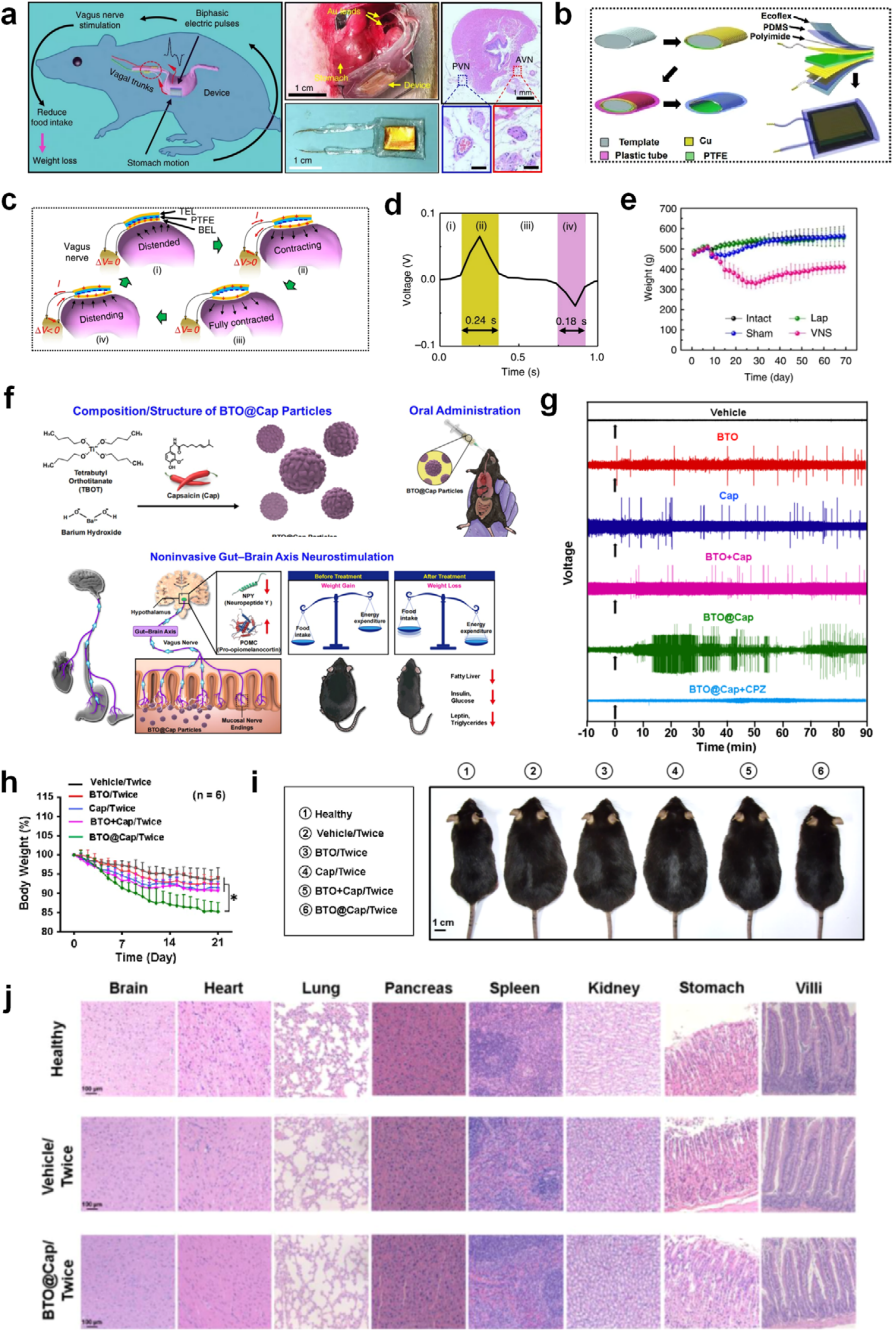
a) Operation principle of the correlated vagus nerve stimulation (VNS) system schematically showing the pathway for biphasic electric signal generation and VNS. b) Schematic fabrication and encapsulation procedures of the VNS device. c) Schematics of the working principle of VNS device under different stomach motion stages. d) A typical single‐cycle voltage biphasic signal corresponding to the four stages of stomach movement at a frequency of 0.05 Hz. e) Weight loss in fully adult rats after implantation of the VNS device. Reproduced under terms of the CC‐BY license.^[^ ^94^ ^]^ Copyright 2018, The Authors, Published by Springer Nature. f) A non‐invasive gut–brain axis neurostimulation system and its operating mechanism. g) Vagus neurograms obtained from test mice subjected to various treatments. The black arrow denotes the initiation of the treatment. h) Daily changes in body weight of test mice subjected to indicated treatments (*n* = 6). i) Representative photographs of test mice that received various treatments, captured at endpoints. j) Histological photomicrographs of major organs retrieved from healthy mice that received a normal chow diet or DIO/10 W mice that received either a vehicle or BTO@Cap particles, two doses per day for a duration of 21 days. Reproduced with permission.^[^ ^95^ ^]^ Copyright 2024, Wiley.

With advancements in nanotechnology, flexible nanoelectrodes are emerging as practical solutions. The triboelectric nanogenerator is a device that harnesses the friction initiation effect and electrostatic induction to convert mechanical energy into electrical energy. This innovation not only addresses the issues of traditional electrical stimulation devices being bulky and cumbersome but also offers self‐sustained stimulation capabilities. An implantable vagus nerve stimulation (VNS) system has been developed based on a flexible triboelectric nanogenerator affixed to the stomach wall of rats. This device generates biphasic electrical impulses in response to stomach wall movement, providing bilateral VNS by wrapping two gold lead wires around the anterior vagus nerve and the posterior vagus nerve near the gastroesophageal junction (**Figure** **10**a).^[^ ^94^ ^]^ To enhance the mechanical robustness and flexibility of the implantable device and to avoid potential corrosion in physiological environments, the entire VNS device is encapsulated in a multilayer film consisting of polyimide, polydimethylsiloxane, and Ecoflex (Figure 10b). When the stomach undergoes peristalsis, the cyclic motion of the triboelectric layers within the VNS device generates electrical signals (Figure 10c,d). Weight loss tests showed that the average body weight of the VNS group was controlled at 410 g, compared to 570–575 g for the other three control groups (Figure 10e). Remarkably, rats in the VNS group were able to return to their normal body weight patterns immediately after the removal of the implanted device.

While results from implantable electrical stimulation devices for the treatment of obesity are promising, challenges remain, such as the risks associated with invasive surgery. To address this, researchers have developed a non‐invasive oral electrical stimulation device. This novel approach combats obesity through oral, self‐powered electrical stimulators consisting of piezoelectric BaTiO_3_ (BTO) particles combined with capsaicin (Cap), designed to activate the vagus nerve (Figure 10f).^[^ ^95^ ^]^ Upon ingestion in diet‐induced obesity (DIO) mice, BTO@Cap particles precisely target and bind to cap‐sensitive sensory nerve endings in the gastric mucosa. In response to gastric peristalsis, these particles generate an electrical signal that travels through the gut–brain axis, ultimately affecting the hypothalamus. By enhancing satiety signaling in the brain, this neuromodulatory intervention reduces food intake and promotes energy metabolism with minimal toxicity. Cervical vagus compound neurograms revealed varying neural responses to Cap, BTO, or Cap+BTO, attributable to Cap's capacity to stimulate the vagus nerve and the mild electrical pulses produced by BTO. Notably, 20 min after administering BTO@Cap, there was a significant increase in neural activity, which was ascribed to the synergistic effects of targeted vagus nerve stimulation by Cap and the mild electrical pulses generated by BTO(Figure 10g). Experimental results indicated that, once the piezoelectric BTO@Cap particles were orally ingested and localized to the cap‐sensitive sensory nerve endings in the gastric mucosa, they effectively functioned as self‐powered VNS neurostimulators. These neurostimulators spontaneously activated the vagal afferent fibers in response to gastric peristalsis, transmitting electrical signals to the brain via the gut–brain axis.

During a 3‐week treatment period, DIO/10 W mice (C57BL/6J mice fed a high‐fat diet for 10 weeks) treated with BTO@Cap pellets exhibited a significant reduction in body weight while maintaining normal locomotor activity compared to control mice (Figure 10h,i). Histological examination of the gastric surface and major visceral organs at the study's endpoint revealed no inflammatory reactions in experimental tissues when compared to those of healthy mice, indicating a lack of significant in vivo toxicity. These findings suggest that oral administration of BTO@Cap is a safe intervention for use in DIO/10 W mice (Figure 10j). Notably, this non‐invasive and easy‐to‐administer approach enhances patient acceptance and adherence, providing strong support for its potential widespread clinical application in treating obesity.

### 3.5 Synergistic Combination Therapies

Obesity is a complex chronic disease influenced by multiple factors, and therefore, combined treatments that target various pathways can yield favorable outcomes. Obese WAT is marked by pathological conditions, such as hypoxia, oxidative stress, inflammation, and altered adipokine secretion, resulting in systemic inflammation and metabolic syndrome (e.g., hyperlipidemia and steatohepatitis), which increase the risk of various adverse health conditions.

Recently, anti‐obesity microneedle patches have been developed for the transdermal and painless delivery of therapeutic agents into subcutaneous WAT of mice, aimed at inducing browning. However, conventional microneedles made of polymer matrices exhibit low drug encapsulation efficiency, while the oxidative stress and chronic inflammation present in obese WAT can hinder browning strategies. This study demonstrates a combined approach by designing a novel microneedle that incorporates manganese dioxide NPs with nanocatalytic properties to mimic SOD and CAT. These NPs scavenge superoxide radicals (O^2‐^) and react with H_2_O_2_ to produce O_2_. Alongside this, Res, a natural antioxidant, is delivered transdermally, reducing oxidative stress and hypoxia while inhibiting inflammation in obese subcutaneous WAT.^[^ ^96^ ^]^

Multifunctional fat‐targeting hybrid NPs (Pep‐PPIX‐Baic NPs) were also developed, combining a fat‐targeting peptide (Fe^3+^), a photosensitizer (protoporphyrin IX), and a browning agent (baicalin). This hybrid NP can induce localized and rapid adipocyte death via PDT and specifically deliver the browning agent into WAT to promote fat browning. Overall, Pep‐PPIX‐Baic NPs demonstrate excellent anti‐obesity potential through the synergistic effects of PDT and fat browning induction, presenting promising nanoplatforms for obesity treatment.^[^ ^97^ ^]^

Additionally, a biomimetic oral H_2_‐producing nanoplatform (HMS/A@GE) has been designed to leverage edible plant‐derived exosomes and H_2_ therapeutics to reverse metabolic dysfunction in T2DM. This platform modulates gut microbiota to mitigate oxidative stress and inflammatory responses, thereby improving insulin resistance and repairing damaged pancreatic tissue.^[^ ^98^ ^]^ Aminoborane serves as an excellent hydrogen donor with acid‐responsive release capabilities. Biocompatible aminoborane has been encapsulated in amino‐modified hollow mesoporous silica, which is then covered with ginger‐derived exosomes, which enhances the biocompatibility of NP system and modulates intestinal flora. Upon reaching the stomach, the system releases substantial amounts of H_2_ under acidic conditions, thereby reducing oxidative stress and inflammation in the liver and pancreas. Moreover, the NPs increase the abundance of *Lactobacillus* spp. and promote the release of indole derivatives, including indole and indoleacetic acid. These indole derivatives activate the intestinal aryl hydrocarbon receptor, promoting the secretion of IL‐22, which helps repair the intestinal mucosal barrier and regulate glycolipid metabolism.

The intragastric satiety‐inducing device (ISD) is another innovative tool designed to promote a sustained feeling of satiety without food intake by applying pressure to the stomach. Endocrine cells in the stomach detect nutrients and secrete appetite‐related hormones, with stimulation of these cells preventing the central nervous system from recognizing hunger. ISDs are closely associated with growth hormone‐releasing peptides (GRP), as the stress induced by the device decreases the secretion of these hormones. To enhance the therapeutic effect of ISDs, these have been combined with PDT by applying a photosensitizer, chlorin e6 (Ce6), to interact with GRP‐secreting cells. This generates ROS, inducing apoptosis in GRP‐producing cells through oxidative stress.^[^ ^99^ ^]^ The combination of these therapies significantly inhibited weight gain compared to treatments with ISD or PDT alone.

## 4 Challenges in Clinical Transformation

Despite these advances, most studies remain in preclinical stages and have not yet translated into widespread clinical applications. There is an urgent need to consider and address several key challenges and issues to achieve clinical translation and ensure long‐term development in this field.

### 4.1 Long‐Term Efficacy

The long‐term efficacy of nanotechnology in the treatment of obesity remains uncertain.^[^ ^100^ ^]^ Although some studies have demonstrated significant short‐term results in animal models, the stability and durability of these treatments in humans require further validation. It is crucial to conduct long‐term clinical studies to determine the sustained effectiveness of nanotechnology in obesity treatment. For instance, although oral NP therapy presents benefits, such as being non‐invasive, low toxicity, and promoting good patient compliance, it remains unclear whether long‐term use may lead to side effects,^[^ ^101^ ^]^ and its effectiveness in managing obesity over extended periods needs clarification through comprehensive clinical observation. Additionally, for NP drug‐delivery systems designed to target adipose tissue, long‐term studies must be conducted to assess their stability and efficacy in diverse populations.

### 4.2 Biosafety

Although nanomaterials have shown significant potential in obesity treatment, the core bottleneck for their clinical translation is safety and biocompatibility. Due to the differences in physicochemical properties, different classes of nanomaterials exhibit unique toxicity mechanisms, immune responses, and metabolic clearance pathways. In terms of toxicity mechanisms, metal nanomaterials are known to induce the generation of reactive oxygen species (ROS). The overproduction of ROS can result in oxidative stress, inflammation, and subsequent damage to proteins, cell membranes, and DNA.^[^ ^102^ ^]^ Concerning immune responses, carbon nanomaterials have been shown to activate lung macrophages, thereby causing inflammation. When these nanomaterials enter the respiratory system, they can be recognized as foreign invaders by the immune cells. The activated macrophages then release pro‐inflammatory cytokines, leading to local and even systemic inflammatory responses.^[^ ^103^ ^]^ Furthermore, upon entering the human body, nanomaterials interact with proteins, leading to the formation of a protein corona. This alters the surface properties of nanomaterials and influences their metabolic behavior within the body.^[^ ^104^ ^]^ For nanomaterials utilized in obesity treatment, these complex biological interactions introduce additional complexity and uncertainty to the interaction between nanomaterials and the biological system. Therefore, biosafety issues should be an important part of nanomedicine research for ultimately clinical translation.

### 4.3 Precise Targeting

Adipose tissue is not uniform in the body but rather dispersed across various “depots”, which complicates the precise localization of adipose tissue. This discontinuity poses a significant challenge for nanotechnology to precisely target adipose tissue during the treatment.^[^ ^105^ ^]^ Currently, the passive targeting strategy through enhanced permeation retention (EPR) effect is unable to adapt to the diverse vascular structure and extracellular matrix between different adipose reservoirs, resulting in uneven accumulation of nanomaterials in the omental fat (with high vascular density) and deep subcutaneous fat, etc.^[^ ^106^ ^]^ At the same time, metabolic activities of adipose tissues, such as lipolysis in the fasting period and lipogenesis in the obesity period, will change the local microenvironment and interfere with the nanomaterials' permeation and cellular uptake. In addition, the interactions between various adipose depots mediated by signaling molecules such as adipokines and exosomes are complex,^[^ ^107^ ^]^ and the existing targeting strategy lacks the ability to achieve spatiotemporal precise targeting using these signaling networks. To address this challenge, more precise targeting methods need to be developed to improve the specificity of nanotechnology for adipose tissue.

### 4.4 Cost and Accessibility

The high costs associated with the development and production of nanotechnology may significantly limit its widespread application in obesity treatment. The design and synthesis of nanomaterials, as well as the development of the delivery systems for these technologies, require complex technologies and specialized equipment, which increases their overall cost. For instance, the processes of creating efficient and safe nanomaterials, surface modification to enhance targeting, and optimizing structures to improve bioavailability all require significant investment in research and development. Furthermore, nanotechnology delivery systems are confronted with technical challenges, including the need for more convenient and effective drug‐delivery methods and improved delivery efficiency, all of which contribute to higher costs.^[^ ^108^ ^]^ Therefore, to increase the utility of nanotechnology in the treatment of obesity, strategies to reduce these costs must be explored.^[^ ^109^ ^]^

Although these challenges may hinder the advancement of nanomedicine, they also inspire us to re‐evaluate future strategies and drive the next wave of nanomedicine development and clinical translation in obesity treatment from multiple dimensions.

## 5 Summary and Prospects

Herein, we provided an overview of the pathological features of obesity, the types of nanomaterials available, and the nanomedicine therapeutic strategies for obesity, aiming to offer valuable references for further research in this field. The application of nanomedicines in the treatment of obesity addresses various pathological processes, including abnormalities in adipose tissue, oxidative stress and inflammation, endocrine and metabolic disorders, and imbalances in gut flora. Commonly used nanoplatforms encompass liposomes, micelles, polymers, metal oxides, AuNPs, carbon‐based nanomaterials, membrane‐coated biomimetic NPs, and cell‐derived EVs.

Treatments for obesity can be broadly categorized into seven types: drug delivery, PDT, thermotherapy, gas therapy, nanoenzymatic catalysis, gut microbial modulation, and electrical stimulation therapy. Over the past decade, the scientific community has witnessed significant advances in the treatment of obesity using nanomaterials. While the application of nanomedicine is still largely in the research phase, it has demonstrated promising potential for improving therapeutic efficacy. To promote the clinical translation of these promising nanomedicine‐based strategies for obesity treatment, several key measures need to be taken.

1) Solutions to the problem of long‐term effectiveness

A systematic validation system is needed to bridge the gap between animal models and human efficacy. A humanized fat metabolism model should be developed to simulate the difference in metabolic rate and immune response in the human body, and combined with dynamic imaging techniques (e.g., PET‐CT to track the distribution of nanomaterials) and continuous monitoring of key biomarkers (e.g., inflammatory factors, such as lipocalin and IL‐6), to comprehensively assess the lasting metabolic regulation of the nanomedicine. Meanwhile, the slow‐release technology of tumor nanomedicines can be borrowed to design core‐shell structured nanoparticles (e.g., TMC‐Zein‐Q system) with controllable release characteristics, which can prolong the duration of action by regulating the drug release kinetics and reduce the risk of weight rebound.

2) Technical approaches for optimizing safety

To address the chronic toxicity risk of nanomaterials, an advance is needed in both the material design and evaluation system dimensions. At the material level, biomimetic modification strategies can be used to reduce toxicity, for example, macrophage membrane‐encapsulated nanoparticles can reduce the liver accumulation rate by mimicking natural immune escape signals, or non‐toxic and non‐immunogenic materials, such as food‐derived materials, can be used as nano‐embedded materials for weight‐loss drugs. In terms of the evaluation system, an organ chip platform based on patient‐derived organoids should be constructed to simulate the long‐term metabolic process of nanomaterials (>6 months) in organs such as the liver and kidney, and combined with single‐cell transcriptome sequencing technology to analyze the effects of nanomaterials on cellular epigenetic regulation, such as mitochondrial function abnormalities or insulin signaling pathway disruption and other potential toxicity mechanisms.

3) Innovative strategies for precisely targeting adipose heterogeneity

The anatomical and molecular heterogeneity of adipose tissue requires “dual precision” in targeting technology. On the one hand, specific enrichment of visceral adipose can be enhanced by dual‐receptor synergistic targeting systems (e.g., SR‐BI/CD36 dual‐targeted liposomes). On the other hand, responsive nanocarriers are designed by utilizing adipose microenvironmental characteristics (e.g., hypoxia, highly reactive oxygen species, or anionic matrix). With the rise of artificial intelligence, a research team has successfully developed highly efficient tumor‐targeting liposomes by screening natural molecules (e.g., wintergreen sapogenin A) with dual functions using AI technology. This approach can be migrated to the treatment of obesity, through the AI rapid screening of molecules that simultaneously target visceral fat markers (such as SR‐BI/CD36) and regulate lipid metabolism, compressing the traditional time‐consuming research and development cycle of several years to a few months, significantly reducing costs, and accurately deciphered the complex targets such as the microenvironment of adipose tissues, which provides a new pathway for the design of highly efficient nano‐delivery systems (e.g., dual‐targeted liposomes).

4) Program for cost control and accessibility implementation

Reducing the cost of nanotherapeutics requires raw material innovation and production process optimization. The production expenses of nanomaterials can be lowered by innovating simpler and more efficient NP preparation techniques. Similarly, enhancing the delivery system of nanotechnology can improve drug delivery efficiency and reduce drug wastage, thereby decreasing treatment costs. Additionally, increased governmental and societal investment in nanotechnology research and development could boost the application of nanotechnology in obesity treatment, improving accessibility and providing effective solutions for more patients.

Looking ahead, we believe that resolving critical scientific issues and technical challenges related to nanomedicine, together with the standardization of preparation methods and evaluation metrics informed by multidisciplinary fields, will allow us to offer more effective, safer, and innovative solutions to the escalating global obesity crisis through nanomedicine. This will facilitate a substantial advancement in the application of nanotechnology within the obesity treatment arena, transitioning from basic research to clinical translation.

**Table 3:** Comprehensive comparison of nanotechnology‐based strategies for obesity treatment.

Types	Modality	Nanomaterial	Delivery method	Target tissue	Mechanism of action	In vivo validation	Refs.
	Drug delivery	Mesoporous silica nanoparticles (MSNs)	Intravenous/Oral	Adipose tissue/liver	High drug loading capacity and controlled release properties to deliver anti‐obesity drugs such as quercetin to inhibit adipogenesis	Reduced fat accumulation and down‐regulation of lipid metabolism gene (PPARγ, FABP4) expression in a mouse model	[31, 32]
Inorganic nanoterials	Thermotherapy	Gold Nanomaterials/Black Scale Nanosheets	Intravenous/Intraperitoneal injection	Adipose Tissue	NIR photothermal conversion disrupts fat cell membranes and releases lipid droplets	Subcutaneous fat was significantly reduced, and metabolic parameters improved in the mouse model	[75]
		Magnetic iron oxide particles	Hypodermic injection	Subcutaneous Adipose tissue	Heat production under alternating magnetic fields induces lipolysis	High‐fat diet mice had reduced fat pads and lower triglyceride levels	[117]
	Gas Therapy	Zn‐Fe micro/nanostructures	Oral	Whole body/adipose tissue	Gastric acid triggers the release of H₂, reducing oxidative stress and inflammation	Increased insulin sensitivity and slowed weight gain in ob/ob mice	[80]
		CeO₂@mSiO₂ nanocomplexes	Intravenous injection	Liver/Adipose tissue	Mimics SOD/CAT activity and reduces ROS levels; promotes macrophage M2 polarization and reduces inflammation	Diabetic mice had a 30% reduction in liver lipids, decreased TNF‐α levels, and improved insulin sensitivity	[84]
	Nanoenzymatic catalysis	Fe₃O₄ NPs	Intraperitoneal injection	Liver/adipose tissue	pH‐dependent enzymatic activity (CAT/POD) activates the AMPK signaling pathway and regulates glycolipid metabolism	Decreased fasting blood glucose and hepatic lipid accumulation in diabetic mice	[85]
	Regulation of the gut microbiota	AgNPs/CrNPs	Oral	Whole body	Increased diversity of gut microbial communities/altered activity of gut flora	Restoration of intestinal flora diversity and reduction of inflammatory factors (LPS, IL‐6) in high‐fat diet mice	[87, 88]
	Electrical Stimulation	Piezoelectric nanoparticles of barium titanateBaTiO3	Oral	Gastric mucosa vagal nerve endings	The piezoelectric effect activates vagal signaling	Reduced body weight in DIO mice, no toxic effects	[95]
Organic nanoterials	Drug Delivery	Liposomes	Subcutaneous/peritoneal injection	WAT/Liver	Surface‐modified targeting molecules (antibodies/peptides/aptamers) for precise drug delivery to adipocytes. Improve drug stability and targeted release to promote WAT browning	WAT browning in obese mice with 10–15% reduction in body weight	[55, 115]
		PLGA microspheres	Hypodermic injection	Subcutaneous WAT	Slow‐release β3‐adrenergic receptor agonist (mirabolone) activates BAT thermogenesis	Mice have reduced visceral fat and increased energy expenditure	[58, 122, 135]
		Third‐generation polyglutamine (P‐G3)	Intravenous injection	Visceral adipose tissue	High charge density interferes with adipocyte lipid storage	Reduced visceral fat accumulation and improved metabolic disorders in mice	[41, 123]
	Photodynamic therapy	AIEgens photosensitizer	Intraperitoneal injection	WAT	Selective targeting of adipocyte lipid droplets and generation of ROS under light triggers a lipid peroxidation chain reaction that induces adipocyte apoptosis and promotes WAT browning	Adipocyte size was reduced, WAT browning marker (UCP1) expression was upregulated, and body weight was significantly decreased in high‐fat diet mice	[73]
	Gas therapy	Peptide Amphiphile Nanogel	Hypodermic injection	Adipose tissue	Slow‐release NO promotes mitochondrial biosynthesis and activates BAT thermogenesis and WAT browning	High‐fat diet mice had slowed weight gain, increased UCP1 expression, and improved glucose tolerance	[81]
	Gas therapy	PLGA NPs	Local injection	Subcutaneous fat	The acid response releases CO₂ to disrupt adipocyte membranes and induce adipocytolysis	The porcine model showed a reduction in subcutaneous fat volume without systemic toxicity	[82]
	Electrical Stimulation	Friction electric nanogenerator (polyimide/gold)	Implantable	Vagus nerve/gastric wall	Gastric peristalsis drives electrical impulses to suppress appetite	21% weight loss in obese rats	[94]
Biomimetic nanoterials	Drug delivery	Macrophage membrane‐coated nanoparticles (rHDL)	Intravenous injection	Inflammatory adipose tissue	Natural immune escape properties of synergistically delivered rosiglitazone and sildenafil promote angiogenesis and WAT browning	CD31 (vascular marker) and UCP1 (browning marker) expression was significantly increased in mouse WAT	[61]
	Photodynamic therapy	Plant exosomes (exosome‐like nanovesicles derived from Oncorhynchus mykiss)	Intraperitoneal injection	Adipose tissue	Natural photosensitizing properties produce reactive oxygen species (ROS) that induce apoptosis in adipocytes; down‐regulate lipid synthesis genes (CEBP, FABP4, PPARG, SREBP)	High‐fat diet mice showed significant weight loss, increased adipocyte apoptosis, and improved glucose metabolism and insulin sensitivity	[74]
	Regulation of the gut microbiota	Plant exosomes	Oral	Intestinal/adipose tissue	Modulates intestinal flora diversity and increases production of SCFAs; appetite suppression via vagus nerve	High‐fat diet mice had 15% lower body weight and improved blood glucose and lipid levels	[89]

## Conflict of Interest

The authors declare no conflict of interest.
